# Sequencing, annotation, and comparative genome analysis of the gerbil-adapted *Helicobacter pylori* strain B8

**DOI:** 10.1186/1471-2164-11-335

**Published:** 2010-05-27

**Authors:** Max Farnbacher, Thomas Jahns, Dirk Willrodt, Rolf Daniel, Rainer Haas, Alexander Goesmann, Stefan Kurtz, Gabriele Rieder

**Affiliations:** 1 Max von Pettenkofer-Institute for Hygiene and Medical Microbiology, Ludwig Maximilian University Munich, Pettenkoferstr. 9a, 80336 Munich, Germany; 2 Center for Bioinformatics, University of Hamburg, Bundesstr. 43, 20146 Hamburg, Germany; 3 Göttingen Genomics Laboratory, Georg-August University Göttingen, Grisebachstr. 8, 37077 Göttingen, Germany; 4 Center of Biotechnology (CeBiTec), University of Bielefeld, Universitätsstr. 27, 33615 Bielefeld, Germany; 5 Current address: Department of Molecular Biology, Division of Microbiology, Paris-Lodron University of Salzburg, Billrothstr. 11, A-5020 Salzburg, Austria

## Abstract

**Background:**

The Mongolian gerbils are a good model to mimic the *Helicobacter pylori*-associated pathogenesis of the human stomach. In the current study the gerbil-adapted strain B8 was completely sequenced, annotated and compared to previous genomes, including the 73 supercontigs of the parental strain B128.

**Results:**

The complete genome of *H. pylori *B8 was manually curated gene by gene, to assign as much function as possible. It consists of a circular chromosome of 1,673,997 bp and of a small plasmid of 6,032 bp carrying nine putative genes. The chromosome contains 1,711 coding sequences, 293 of which are strain-specific, coding mainly for hypothetical proteins, and a large plasticity zone containing a putative type-IV-secretion system and coding sequences with unknown function. The *cag*-pathogenicity island is rearranged such that the *cag*A-gene is located 13,730 bp downstream of the inverted gene cluster *cag*B-*cag*1. Directly adjacent to the *cag*A-gene, there are four hypothetical genes and one variable gene with a different codon usage compared to the rest of the *H. pylori *B8-genome. This indicates that these coding sequences might be acquired via horizontal gene transfer.

The genome comparison of strain B8 to its parental strain B128 delivers 425 unique B8-proteins. Due to the fact that strain B128 was not fully sequenced and only automatically annotated, only 12 of these proteins are definitive singletons that might have been acquired during the gerbil-adaptation process of strain B128.

**Conclusion:**

Our sequence data and its analysis provide new insight into the high genetic diversity of *H. pylori*-strains. We have shown that the gerbil-adapted strain B8 has the potential to build, possibly by a high rate of mutation and recombination, a dynamic pool of genetic variants (e.g. fragmented genes and repetitive regions) required for the adaptation-processes. We hypothesize that these variants are essential for the colonization and persistence of strain B8 in the gerbil stomach during in ammation.

## Background

*Helicobacter pylori *is a Gram-negative human pathogen that colonizes the gastric mucosa of about half of the world population. The majority of carriers develop an asymptomatic chronic gastritis that persists for decades. In up to 20% of the *H. pylori*-infected people severe diseases are developed such as peptic ulcer, gastric adenocarcinoma, and MALT (mucous-associated lymphoid tissue)-lymphoma [1]. Epidemiological studies reveal a high prevalence of *H. pylori *in malignant gastric diseases. Therefore, in 1994, the WHO declared *H. pylori *as carcinogen of the class I (definitive) [2]. Only about 1% of *H. pylori*-infected humans develop malignant gastric sequelae, thus indicating a multi-factorial process that includes host factors (gene polymorphisms) [3], environmental factors (alcohol and nicotine abuse, diet etc.) [4], and bacterial factors. Beside others, two major *H. pylori *virulence factors intensively studied in this respect are the vacuolating cytotoxin VacA and the cytotoxin-associated antigen CagA. After secretion VacA acts as a multifunctional toxin causing alterations in late endosomes and mitochondrial membrane permeability [5]. Furthermore, VacA inhibits T-cell proliferation via β2-integrins, supporting the chronicity of *H. pylori *infection [6]. CagA was just recently shown to be an oncoprotein based on the observation that *cag*A-transgenic mice develop significantly increased neoplasia [7]. The *cag*A gene is part of the *cag*-pathogenicity island (*cag*-PAI), consisting of about 30 genes. These genes encode a type IV-secretion system (T4SS), a needle-like apparatus at the surface of the pathogen translocating the effector protein CagA into the host cells. The injected CagA protein becomes tyrosine-phosphorylated by the host kinases *Src *and *Abl *[8]. The T4SS and CagA proteins are involved in numerous signalling cascades associated with cell proliferation, motility, actin cytoskeletal rearrangements, disruption of cell-to-cell junctions, pro-inflammatory responses and suppression of apoptosis [9]. Thus, it is now clear that the *cag*-PAI encoded virulence apparatus plays a pivotal role in *H. pylori *pathogenesis.

Several animal models were tested for *H. pylori *colonization, persistence, and pathogenesis. Although the frequently used mouse model comes with a large reservoir of genetic tools such as specific transgene and knock-out mouse lines, its major disadvantage should not be neglected, as mice so far cannot persistently be infected with *H. pylori *type I-strains expressing a functional T4SS. The stability of the *cag*-PAI is lost in mice over time of infection [10]. The Mongolian gerbil animal model is better mimicking the human situation and is very suitable to investigate the role of the major *H. pylori *virulence factors on the onset and process of gastric carcinogenesis. In 1998 Watanabe *et al*. first demonstrated that *H. pylori*-infected Mongolian gerbils develop gastric cancer after 62 weeks of infection with a prevalence of 37% [11]. This even occurs without adding any co-carcinogens. Using the gerbil-adapted *H. pylori *type I-strain B128, originally isolated from the human stomach of a peptic ulcer patient, several groups showed that this pathogen successfully colonizes the gerbil stomach over time [12,13]. After eight weeks of infection a severe antral and corpus gastritis is induced, followed by a precancerous process of atrophy, metaplasia, and dysplasia as earlier defined by the pathologist Correa [14]. Less virulent *H. pylori*-strains with a defective T4SS, so called type II-strains, do not proceed in a corpus-dominant atrophic gastritis, a risk factor for developing gastric adenocarcinoma. Thus, an early inflammation later results in the gastric cancer pathway, which strictly depends on a functional T4SS in the Mongolian gerbil model.

*H. pylori *is known for its remarkably high level of genetic diversity creating a dynamic pool of genetic variants. However, it must also maintain its genomic integrity. Kang and Blaser (2006) proposed that this pool of genetic variants delivers a sufficient genetic diversity to allow *H. pylori *to occupy all the potential niches in the stomach (for example, antrum and corpus mucosa) [15]. The usual diversification mechanism involves a frequent intraspecific recombination [16] and an increased mutation rate [17], but this is actually not enough to explain the extreme genetic diversity of *H. pylori*. Additionally, the large amount of repetitive DNA sequences observed in previously available *H. pylori *genomes, supports this remarkable diversification phenomenon. In particular, homopolymeric nucleotide stretches or di- and oligonucleotide repeat tracts can be phase variable expressed by the regulatory mechanism of slipped strand mispairing (ssm) [18-20]. Non-random distribution of long regions of nucleotide identity thousands of base pairs apart (i.e. repeats) may serve to enhance programmed rearrangements and genetic diversity in *H. pylori*, which appears to be a highly conserved mechanism in prokaryotes [19].

The *com*B-system, a modified T4SS, enables *H. pylori *to take up exogenous DNA by natural competence. This allows such DNA to be incorporated into the genome through homologous recombination [21]. Since in many cases the human stomach is colonized with several different *H. pylori*-strains, a potential recombination within all individuals of this species might allow a panmictic population structure [22]. However, despite extensive microdiversity, *H. pylori *strains are fundamentally similar to each other in overall gene content and organization. Applying molecular typing techniques like the multilocus sequence typing (MLST), using the polymorphisms of seven housekeeping genes, it was shown that genetic similarity is conserved in *H. pylori *strains from distinct geographical regions [23,24]. The migration of nations as well as the slave trade between Africa and America is consistent with the prevalence of *H. pylori *populations distributed within these humans [25,26].

*H. pylori *was the first species of which two complete genomes were sequenced [27,28]. These were subject to a comparative analysis elucidating the molecular mechanisms regarding the pathogenicity and virulence of bacteria originating from patients with different gastrointestinal diseases (strain 26695 originates from patients suffering from a chronic gastritis and strain J99 originates from patients with duodenal ulcer).

Both genomes contain about 1.6 Mbp. Pairs of ortologuous genes show a sequence identity of about 93% on the nucleotide level, and several inversions and transpositions become apparent when comparing the entire genomes. The two genomes have about 1,400 core genes in common, while 7% of the coding sequences are strain-specific, mainly located on hypervariable regions, called plasticity zones (PZ) [29]. Up until now, another seven fully sequenced and annotated genomes of *H. pylori*-strains HPAG1 [30], shi470 [31,32], G27 [33], HPKX_438_AG0C1 and HPKX_438_CA4C1 [34] as well as P12 (NC_011498, unpublished) and HPB38 (NC_012973, unpublished) became available for further comparative analyses.

Recently, another two *H. pylori *strains isolated from patients with gastric cancer (98-10) and from patients with gastric ulcer (B128) were sequenced and their 51 and 73 supercontigs, respectively, were compared for identifying strain-specific genes [35]. *H. pylori *B128 is the parental strain that was subsequently gerbil-adapted. Here we present the whole genome analysis of the gerbil-adapted *H. pylori *strain B8 that originates from *H. pylori *strain B128, but was adapted to Mongolian gerbils by several subculturing steps and stomach passages of up to four weeks. This gerbil-adapted strain B8 is a typical type I-strain able to induce severe gastritis as well as gastroduodenal sequelae over time [36,37].

At first, we considered some basic features of the genome of *H. pylori *strain B8, including an analysis of the repeats. Second, we looked at the similarities and differences of the genome sequences and proteomes of strain B8 and B128, paying special attention to the missing and incomplete coding sequences due to the fact that the genome sequence of the *H. pylori *strain B128 is not closed yet. Third, we compared the whole genome of strain B8 with other fully sequenced *H. pylori *strains. Although the other strains are not directly related to strain B8, it is interesting to compare the new whole genome sequence of *H. pylori *strain B8 to other completely sequenced and well-annotated strains, to study the genetic diversity of *H. pylori*. Finally, we attempted to identify candidates for strain-specific coding sequences that may be associated with the adaptation of strain B8 to the stomach of the Mongolian gerbil.

## Results

### General features of the genome of *H. pylori* strain B8

The whole-genome sequencing of *Helicobacter pylori *strain B8 was done by a combination of Sanger sequencing (coverage 2.5×) and pyrosequencing technologies (454-sequencing, coverage 16×). The remaining gaps were closed by PCR and combinatorial multiplex PCR on isolated genomic DNA as well as by primer walking on recombinant plasmids. We also applied Sanger technology for resequencing all length variable genes, which in turn improved the sequence quality. All in all, our approach resulted in a continuous high quality sequence. The genome of strain B8 was deposited in DDBJ/EMBL/Genbank on December 1, 2009 and has accession number FN598874. The plasmid of strain B8 was deposited in DDBJ/EMBL/Genbank on January 26, 2010 and has accession number FN665651.

**Figure 1:**
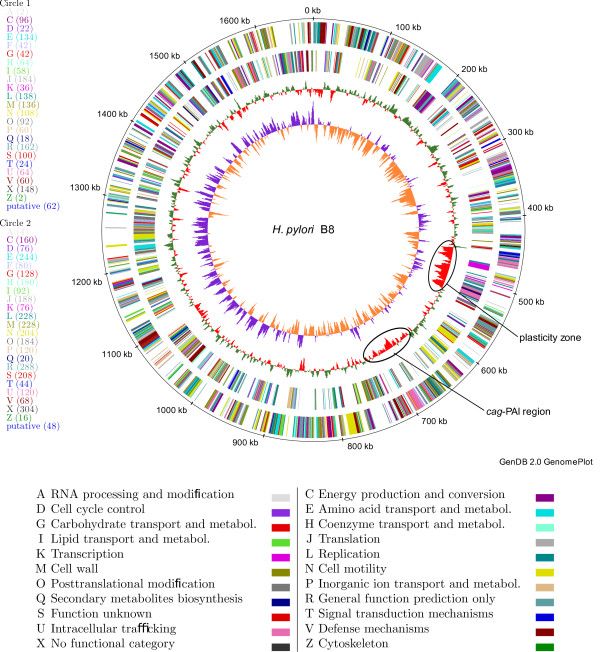
**Circular plot of the chromosome of strain B8**. From outside to inside the circles show (a) coding sequences on the forward strand, colored according to the COG category whose code and meaning is given in the table below the plot, (b) coding sequences on the reverse strand, colored according to the COG category, (c) average GC content, calculated for a window of 3,000 bp shifted by 1,000 bp over the genome sequence in each step, (d) GC-skew (*G *+ *C = G C*), calculated for a window of 3,000 bp, shifted by 1,000 bp over the genome sequence in each step. For better readability (c) and (d) are scaled up. For each COG category the number of genes belonging to it is shown in brackets in the left legend. For each COG category, the bottom legend shows the letter code, the meaning, and the color of the categories used in the circle plot. The *cag*-PAI and the plasticity zone are marked by an ellipse on the second inner circle. The plot was created by the software GenDB.

The genome of strain B8 consists of a circular chromosome of 1,673,997 bp with a GC content of 38.8% and 1,711 coding sequences, of which 929 (54.3%) are functionally annotated (Table 1 and Figure 1). There are 496 conserved hypothetical genes in strain B8. The circular genome was split such that the *ori*C starts at the first position of the genome (Figure 1). The genome comes with a plasmid, named pHPB8, consisting of 6,032 bp with a GC content of 35.9% and nine coding sequences, five of which are functionally annotated.

**Table 1:** General features of different *H. pylori *genomes.

*H. pylori * strain	B8	26695	J99	HPAG1	P12
accession number	FN598874	NC_000915	NC_000921	NC_008086	NC_011498
**Basic Features**					

chromosome length	1,673,997 bp	1,667,867 bp	1,643,831 bp	1,596,366 bp	1,673,813 bp

plasmid	pHPB8 (6,032 bp, GC 35.9%)			pHPAG1 (9,370 bp, GC 36.4%)	pHPP12 (10,225 bp, GC 35.1%)

GC content	38.8%	38.9%	39.2%	39.1%	38.8%

CDS density	91.8%	90.2%	90.2%	91.9%	89.7%

number of CDS	1,711	1,576	1,489	1,536	1,568

average length	897	955	990	954	957

annotated	929 (54.3%)	918 (58.2%)	712 (47.8%)	1,013 (66.0%)	1,111 (70.9%)

strain-specific CDS	293	204	112	152	202

thereof annotated	3	9	1	8	2

**Type IV Secretion Systems**^#^					

*cag*PAI	670,637 - 720,370 HPB8_696 - HPB8_741	+	+	+	552,705 - 589,225 HPP12_0527 - HPP12_0555

*com*B	I: 1,575,414 - 1,578,323 HPB8_1608 - HPB8_1610*	+			I: 13,587 - 16,496 HPP12_0013 - HPP12_0015
			
	II: 1,551,606 - 1,554,461 HPB8_1583 - HPB8_1585*		II: 37,867 - 40,719 jhp0034 - jhp0036	II: 38,692 - 41,645 HPAG1_0036 - HPAG1_0039	II: 36,339* -* 40,378 HPP12_0033 - HPP12_0037

T4SS-3 (*tfs*3)	510,833 - 526,789 HPB8_538 - HPB8_554*	fragmented	-	-	1,394,833 - 1,411,026 HPP12_1320 - HPP12_1337

T4SS-4 (*tfs*4)	fragmented, surrounding T4SS-3		?	?	452,423 - 492,710 HPP12_0437 - HPP12_0473

**Plasticity Zones**^#^					

	PZ1:452,011 *-* 533,220 HPB8_481 - HPB8_564	left: 449,150 - 479,531 HP_0428 - HP_0460	I: 1,012,090 - 1,057,038 jhp0914 - jhp0951		PZ1:452,423 *-* 492,710 HPP12_0437 - HPP12_0473
			
		right: 1,044,552 - 1,071,068 HP_0980 - HP_1009			PZ2: 1,043,356 - 1,053,784 HPP12_0980 - HPP12_0993
					
					PZ3: 1,394,833 - 1,423,818 HPP12_1320 - HPP12_1353

**RNA Elements**^§^					

rRNA 23S | 16S | 5S	2 | 2 | 2	2 | 2 | 2+1^‡^	2 | 2 | 2	2 | 2 | 2	2 | 2 | 2

tRNA	36	36	36	36	36

# notation for nucleotide range: *first position last position*; notation for gene range: *first_gene-last_gene*

* genes occur on reverse strand

§ number of gene copies

‡ strain 26695 contains two sets of 23S and 5S rRNA and one additional 5S rRNA without associated 23S rRNA

### Analysis of repeats in the complete genome of strain B8

Repeats can occur in a coding sequence (e.g. in the *cag*Y-gene) or in form of duplicated genes (e.g. *vir*B-genes) or duplicated sequence regions somewhere in the genome. There are 144 repeats with a length of at least 100 bp and at least 80% sequence identity (Table 2). The repeats have lengths of up to 4,381 bp, and 49 of the 144 repeats are inverted repeats for which the second instance of the repeat occurs on the reverse strand. Especially the *vir*B-genes show homology in different repeat regions, e.g. they are located on the *cag*-PAI and the plasticity zone (PZ) of strain B8. Furthermore, restriction endonuclease genes are located at different loci on the genome.

**Table 2:** List of the 43 most significant repeats in the genome of strain B8, ordered by increasing E-value.

length in bp	position	strand	length in bp	position	number of differences	E-value	sequence identity in %
3241	352423	+	3242	646346	2	0.00e + 00	99.94

818	1614435	+	819	1615640	94	0.00e + 00	88.52

801	1490715	+	802	1543145	6	0.00e + 00	99.25

648	984653	+	647	1039045	1	0.00e + 00	99.85

4381	447896	-	4384	1528769	30	0.00e + 00	99.32

2059	287098	+	2060	912424	4	0.00e + 00	99.81

1164	396381	-	1165	832923	11	0.00e + 00	99.06

• 2207	96959	+	2201	323043	39	0.00e + 00	98.23

2439	210771	+	2438	628615	4	0.00e + 00	99.84

563	690091	+	563	690481	11	0.00e + 00	98.05

588	273255	+	588	1414805	11	0.00e + 00	98.13

• 1135	590750	+	1134	1083761	79	0.00e + 00	93.04

2213	580672	-	2213	901965	7	0.00e + 00	99.68

508	715518	+	514	720788	14	1.88e - 259	97.28

799	692230	+	796	692782	109	4.26e - 247	86.36

663	130801	-	671	1311865	104	1.76e - 186	84.50

602	167233	+	602	869141	87	8.22e - 176	85.55

425	272770	+	425	1414201	32	1.54e - 170	92.47

396	351462	-	395	1070874	28	1.56e - 161	92.93

298	445088	-	298	1538257	9	1.06e - 143	96.98

398	131808	-	401	1311134	40	1.08e - 142	90.02

282	447722	-	280	1533034	11	7.50e - 130	96.10

421	496148	-	424	526803	61	6.03e - 121	85.61

276	627392	+	277	627526	16	1.58e - 116	94.22

406	130325	-	406	1312519	58	5.69e - 116	85.71

205	213298	+	207	631421	2	4.58e - 107	99.03

468	692461	+	474	693450	91	5.98e - 103	80.80

308	444293	-	316	1539031	40	6.68e - 96	87.34

233	692119	+	233	692236	15	3.32e - 93	93.56

213	85440	-	216	468947	11	2.05e - 91	94.91

232	692120	+	232	692789	16	1.05e - 90	93.10

279	105830	+	279	1670378	32	9.03e - 89	88.53

225	1437117	-	226	1456289	18	1.55e - 83	92.04

243	693340	+	244	693664	24	5.80e - 83	90.16

394	691841	+	396	692394	77	8.27e - 83	80.56

191	397488	-	193	832659	9	3.26e - 82	95.34

264	667779	-	263	1052938	31	3.39e - 82	88.26

319	130360	-	318	630425	51	1.95e - 80	84.01

319	130360	-	318	212583	51	1.95e - 80	84.01

264	798608	+	267	800003	34	6.00e - 79	87.27

323	691912	+	323	693019	54	4.38e - 78	83.28

367	913847	+	367	1312534	70	7.92e - 78	80.93

In particular, all repeats of minimum length 100 with at least 80% sequence identity are shown. The length and the position of the first and second instance of the repeat are shown in column 1 and 2 and in column 4 and 5. The strand of the repeat is given in column 3. Column 6 shows the number of differences (i.e. insertions, deletions, replacements) in an optimal alignment of the two repeat instances. Column 7 shows the E-value of the repeat and column 8 the sequence identity. The two bullets mark the repeats also occurring in all four *H. pylori *reference strains 26695, J99, HPAG1, and P12.

Altogether, 4.3% of the whole genome of strain B8 is covered by repeated sequences. This repeat density is similar for three of the genomes of the other *H. pylori *strains: using the same parameters as above, one obtains a repeat density of 4.5% for strain J99 (187 repeats), 4.5% for strain HPAG1 (185 repeats), and 4.1% for strain P12 (160 repeats). Only strain 26695 has a remarkably higher repeat density of 5.9% (207 repeats). While *H. pylori *is considered to be a very repetitive bacterial species [19,38], the repeat densities of the different strains are not remarkably high when compared to all other bacterial genomes: The distribution of repeat densities over 1,052 bacterial genomes achieves a median of 4.0% and an average of 4.6% (Additional file 1, Figure S1). For example, there are 463 bacterial genomes with a repeat density of more than 4.3%.

To find common repeats, a blastn comparison of the repeats of the five *H. pylori *strains B8, 26695, J99, HPAG1, and P12 was performed. We consider a repeat to occur in another genome if there is an 80/80 blastn hit of this repeat to any repeat in the set of repeats of this genome. We say that there is an 80/80 blastn hit between two repeats if there is a blastn between any of the four pairs of repeat instances from the two repeats which has at least 80% sequence identity and covers at least 80% of both instances. Strain B8 contains 51 repeats occurring in any of the other *H. pylori *strains. More specifically, there are 21 repeats occurring in only one other strain, eight occurring in two other strains, two occurring in three other strains, and two occurring in all other strains. Interestingly, these last two repeats (see Table 2, rows marked by a bullet) are very long repeats of 2,201 and 1,134 bp, respectively. The left instance of the 2,201 bp repeat partly overlaps with gene HPB8_96 and the 16s rRNA HPB8 r1, while the right instance occurs in a region with no functional element. Both instances of the 1,134 bp repeat contain the coding sequence for the outer membrane protein Omp22. This is only annotated as such in one instance in strain 26695, but not in the other three strains. Strain B8 has 16 repeats occurring in strain 26695, 15 repeats occurring in strain J99, 13 repeats occurring in strain HPAG1, and 7 repeats occurring in strain P12.

### Comparative analysis of the coding sequences of strains B8 and B128

To identify genes involved in gerbil-adaptation, a comparative genome analysis of strain B8 with the original strain B128 was conducted. The available sequence of strain B128 consists of 73 supercontigs. All supercontigs were mapped to the whole genome of strain B8. Additional file 1, Table S1 lists the specific positions and the quality of the mapping. All supercontigs can be mapped to the genome of strain B8. In total, about 98% of the whole genome sequence of strain B8 is covered by B128-supercontigs. Due to some overlaps (of lengths between 2 and 293 bp) of the mapped supercontigs (Additional file 1, Table S2), seven of these may be fused resulting in 66 B128-supercontigs. The resulting 65 gaps are between 1 and 4,608 bp long.

For further analysis, e.g. identification of specific gerbil-adapted genes, a list of genes of strain B8 not completely covered by B128-supercontigs was compiled (Additional file 1, Table S3). This list allows to identify "weak" B8-singletons, i.e. genes which have the singleton-property due to the fact that the genome of strain B128 has gaps. Our comparison is based on 80/80 blastp hits, i.e. blastp hit of at least 80% sequence identity covering at least 80% of the protein sequence. A gene is regarded as a singleton if there is no 80/80 blastp hit of the protein sequence in the set of all proteins of the reference genomes. The set of genes of a reference strain with an 80/80 blastp hit in every other strain is referred to as the core genome.

All B8-singletons appearing completely within the covered regions of strain B8 are called "strong" B8-singletons. The uncovered regions of strain B8 contain 35 kbp and include 60 genes. 33 of these genes completely occur with at most 2% differences (i.e. insertions, deletions, and replacements) somewhere else in the B128-supercontigs, or there is an 80/80 blastn hit, i.e. a blastn hit of at least 80% sequence identity covering at least 80% of the length of the coding sequence. This and the large amount of repeats suggest a possible reason why the gaps in the genome of strain B128 (which was purely sequenced using 454-sequencing) were not closed: The 454-reads may have been too short to give enough evidence for assembling regions containing duplicated genes or repeated regions in the genome of strain B128.

Further comparative analysis of the genome of strain B8 with B128 reveals that altogether there are 1,652 of 1,711 coding sequences (i.e. 96.6%) of strain B8 matching completely with at most 2% differences to the B128-supercontigs. That is, there is an alignment of the complete coding sequence (i.e. from the first to the last position) and a substring of the B128-supercontigs with at most 2% differences. The percentage refers to the length of the coding sequence. Table 3 lists the distribution of the difference values in the best complete matches (i.e. matches with minimum number of differences) of each coding sequence to the B128-supercontigs. For example, there are 1,281 coding sequences in strain B8 matching exactly (with no differences), 269 coding sequences matching with one difference, and thus 1,550 coding sequences matching with at most one difference.

**Table 3:** Distribution of nucleotide differences in the best matches of the coding sequences of strain B8 against the supercontigs of B128.

number of nucleotide differences	number of CDS	cumulative number
0	1281	1281

1	269	1550

2	64	1614

3	16	1630

4	10	1640

5	6	1646

7	2	1648

9	1	1649

12	1	1650

15	1	1651

40	1	1652

The second column shows the number of sequences matching the number of differences. The third column accumulates the numbers of the second column.

### Comparative genome analysis of the proteome of strains B8 and B128

Comparing the B8-proteome to the B128-proteome reveals that there are 425 amino acid sequences in strain B8 such that there is no 80/80 blastp hit in the B128-proteome, see Additional file 1, Table S4, row marked by a bullet. Among these singletons there are 371 singletons (i.e. 87%), for which the corresponding coding sequence has a complete match with at most 2% differences in the B128-supercontigs, see Additional file 1, Table S5 for a complete list. From these numbers one concludes that for many genes the DNA sequence is present in the B128-supercontigs, but the corresponding gene has not sufficiently been annotated in strain B128. The remaining 54 singletons of 425 protein sequences are given in Additional file 1, Table S6. Out of the 54 singletons, 42 are completely or partly located in the regions not covered by the B128-supercontigs and therefore have to be regarded as "weak" (putative) singletons (Additional file 1, Table S6, green). The remaining 12 "strong" (definitive) singletons are listed in Table 4. Additional file 1, Table S4 gives the numbers of singletons obtained when varying the minimum required sequence identity and coverage of the blastp hits in the range from 70-100%. The ratio of the number of singletons and the number of coding sequences was comparable over the whole range, indicating a similar quality of the nucleotide sequence.

**Table 4:** List of 12 singletons of strain B8, i.e. genes which completely occur outside of the uncovered regions and have neither an 80/80 blastp hit in the B128 proteome nor a complete match with at most 2% differences on the DNA level.

	gene	product	J99-ortho	26695-ortho
1	HPB8_138	periplasmic protein TonB		

2	HPB8_277	hypothetical protein predicted by Glimmer/Critica		

3	HPB8_399	conserved hypothetical protein		HP1105

4	HPB8_639	hypothetical protein predicted by Glimmer/Critica		

5	HPB8_655	Hydrogenase expression/formation protein hypD2		

6	HPB8_692	Plasminogen-binding protein pgbA		

7	HPB8_888	ferrous iron transport protein B	jhp0627	HP0687

8	HPB8_922	conserved hypothetical protein	jhp0654	HP0716

9	HPB8_976	hypothetical protein predicted by Glimmer/Critica		

10	HPB8_1447	conserved hypothetical protein		HP1187

11	HPB8_1483	methyl-accepting chemotaxis protein	jhp0075	HP0082

12	HPB8_1618	hypothetical protein predicted by Glimmer/Critica		

The last two columns show the locus tags of all genes in strain J99 and strain 26695 which are ortholog to the given gene of strain B8.

There are 49 coding sequences in strain B128 such that the corresponding proteins do not have an 80/80 blastp hit in the B8-proteome (Additional file 1, Table S7). Most of these B128-proteins are classified as singletons due to genetic phase variation, e.g. earlier or later stop codon. In general, these B8- and B128-singletons are of interest in analyzing the gerbil-adaptation process leading from strain B128 to strain B8 (see Discussion).

A comparison of the complete *cag*-PAI of the strains B8 and B128 was not possible because the B128-sequence has several gaps in the *cag*-PAI region. Nevertheless, it was possible to compare the two major virulence factors CagA and VacA of the two strains. Both factors show 100% identity on nucleotide and protein level.

### Comparison of the genomes of strain B8 and other *H. pylori* strains

The chromosome of strain B8 (1,673,997 bp) is longer than the chromosomes of strains 26695 (1,667,867 bp), J99 (1,643,831 bp), HPAG1 (1,596,366 bp), and P12 (1,673,813 bp). Strain B8 has 1,711 coding sequences with an average size of 858 bp, see Table 1. The average size is smaller than in the other strains. The phase variation of genes is one reason for the high genetic diversity observed in the available *H. pylori *genomes [39]. This is represented in the small average size but large number of genes in strain B8. In total, we found 52 genes (i.e. 3% of all genes) of strain B8 with a length variation mainly due to gene fragmentation (see Discussion). Furthermore, the density of the coding sequences in strain B8 is still relatively high: 91.8% of the chromosome is covered by coding sequences. This is higher than the coding density for strain 26695 (1,576 genes and 90.2% coding density), for strain J99 (1,489 genes and 90.2% coding density) and for strain P12 (1,568 genes and 89.7% coding density). Only strain HPAG1 has a slightly higher coding density (1,536 genes and 91.9% coding density).

### Singletons of strain B8

To identify strain-specific genes (singletons) we used the software tool EDGAR [40], whose comparison model is based on pairwise comparisons of protein sequences using blastp [41].

**Figure 2:**
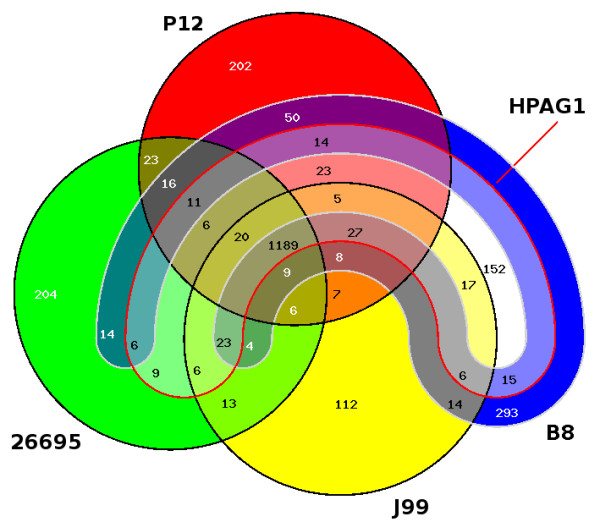
**Venn-diagram of the genomes of the *H. pylori *strains 26695, J99, HPAG1, P12, and B8**. The diagram shows the numbers of orthologous coding sequences of these strains. The genes of the core genome (1,189) are present in all strains. Singletons: 202 coding sequences of strain P12 (red), 204 coding sequences of strain 26695 (green), 112 coding sequences of strain J99 (yellow), 152 coding sequence of strain HPAG1 (white), and 293 coding sequences of strain B8 (blue) have no orthologs in the other four genomes. The diagram was drawn by the software EDGAR.

A comparison of the chromosomes of strains 26695, J99, HPAG1, P12, and B8 gives a core genome of 1,189 genes. 293 coding sequences of strain B8 are strain-specific (see Figure 2 and Additional file 1, Table S8 for a complete list). Of these, 57 are functionally annotated, 42 are conserved hypothetical, and 194 are hypothetical genes. 36 singletons are located in the PZ of strain B8. Interestingly, the other four strains have considerably less singletons: strain HPAG1 has 152 singletons, strain J99 has 112 singletons, strain 26695 has 204 singletons, and strain P12 has 202 singletons. The larger number may be due to the fact that strain B8 is adapted to the Mongolian gerbil while the others are not. In contrast, when using less strict blastp hits with minimum bit score of 100, one obtains 144 singletons.

Among the 293 strain-specific coding sequences, 57 are functionally annotated. Interestingly, the typical genes related to DNA modification e.g. DNA methylases (HPB8_1059, HPB8_1100, HPB8_1101, HPB8_1103, HPB8_1538, and HPB8_1592) and restriction endonucleases (HPB8_1060, HPB8_1119, HPB8_1120, HPB8_1121, and HPB8_1706) are present in the genome of strain B8 (Additional file 1, Table S8). Furthermore, we found two genes coding for proteins enabling DNA transfer (HPB8_485, HPB8_492, and HPB8_493), a putative transposase (HPB8_518), and two CDP hydrolases (HPB8_1081 and HPB8_1082).

We analyzed the singletons of strain B8 according to the pathogenicity of the strains. To do so, we compared the whole genome of strain B8 with the genomes of the duodenal ulcer strains J99 and P12 as well as the gastritis strains 26695 and HPAG1. In the case of the duodenal ulcer strains and gastritis strains we obtained 35 and 74 singletons of strain B8 in addition to the 293 singletons, respectively (Additional file 1, Tables S9 and S10). A remarkably high number of singletons of the comparison to the gastritis strains belong to the group of T4SS proteins (VirB and VirD). These are located within the *tfs*3 on the PZ3 of the duodenal ulcer strain P12 and therefore are missing in Additional file 1, Table S9.

### Description of the plasticity zone of strain B8

**Figure 3:**
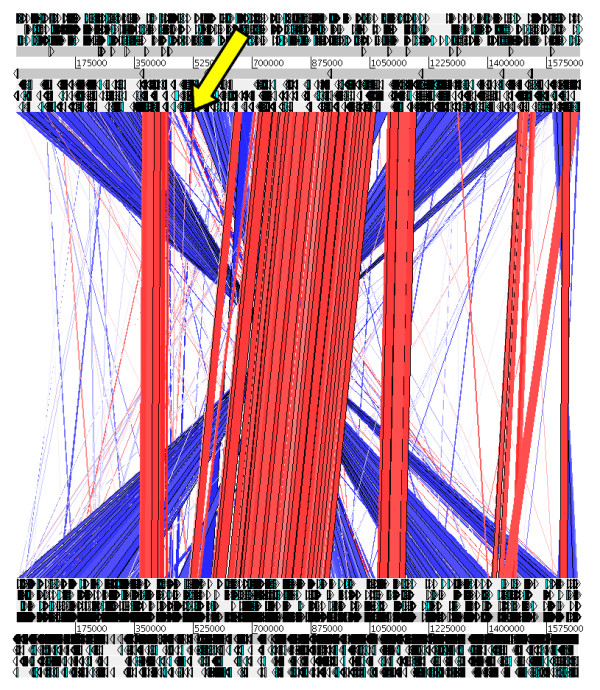
**Synteny plot of *H. pylori *strain B8 (top) vs. strain 26695 (bottom)**. The plot is based on pairwise blastn hits (blast version 2.2.21) with the following parameters: E-value: 10, minimum sequence identity: 80%, minimum bit score: 80. Red indicates homologous regions. Blue indicates inverted homologous regions. White indicates regions without homology in the other genome. The yellow arrow marks the PZ of strain B8. The graph was created by the Artemis Comparison tool.

**Figure 4:**
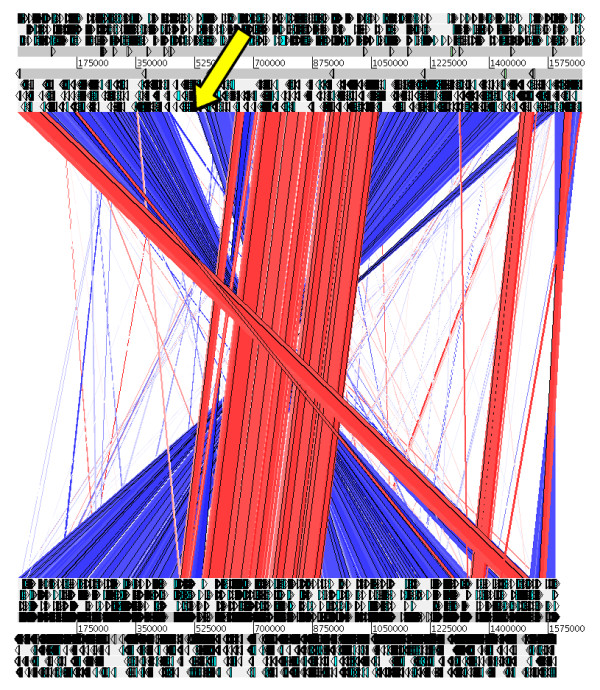
**Synteny plot of *H. pylori *strain B8 (top) vs. strain HPAG1 (bottom)**. The plot is based on pairwise blastn hits (blast version 2.2.21) with the following parameters: E-value: 10, minimum sequence identity: 80%, minimum bit score: 80. Red indicates homologous regions. Blue indicates inverted homologous regions. White indicates regions without homology in the other genome. The yellow arrow marks the PZ of strain B8. The graph was created by the Artemis Comparison tool. The synteny plots of strain B8 versus strains J99 and P12 can be found in Additional file 1, Figures S3 and Figure S4.

The plasticity zone of strain B8 is located between position 452,011 and position 533,220, i.e. it consists of 81,210 bp (Figure 1; yellow arrow in Figures 3 and 4). Thus it is longer than the PZs of each of the genomes strain 26695, J99, HPAG1, and P12. The PZ of strain B8 contains 84 coding sequences (HPB8_481 to HPB8_564), see Table 1. It has a lower GC-content (34%) than the overall genome. For the *H. pylori *strains 26695 and J99 it was previously shown that the PZs are flanked by the *fts*Z-gene and the 5S-/23S-rRNA gene pair [27,28]. This also holds for strain B8, where the *fts*Z-gene has the locus tag HPB8_566. In the PZ of strain B8 typical genes are present (Additional file 1, Table S11): (a) *top*A (occurring twice as HPB8_487 and HPB8_537) encoding a DNA topoisomerase I, (b) a conserved hypothetical protein (HPB8_501) containing a *vir*D2 relaxase domain, (c) *par*A (HPB8_505) encoding a putative chromosome partitioning protein, (d) *orf*Q (HPB8_506) containing domains characteristic for DNA methylases and helicases [42], and (e) the integrase/recombinase coding gene *xer*D (HPB8_556). The PZ of strain B8 also contains a transposable element ISHp608 from position 490,732 to 488,901. The type IV-secretion system *tfs*3 is located from position 510,833 to 526,789 (HPB8_538 to HPB8_554). It is surrounded by a partial *tfs*4 (Table 1).

In the genome of strain J99, the PZ contains two genes jhp0947 and jhp0949 that are reported to be associated with gastric diseases [43-45]. Strain B8 contains a coding sequence HPB8_512 with homology to jhp0947 and a coding sequence HPB8_514 with homology to jhp0949. HPB8_512 and HPB8_514 are both located in the PZ of strain B8. There is also a coding sequence HPB8_506 with homology to jhp0927, a coding sequence HPB8_474 with homology to jhp0960, and a coding sequence HPB8_473 with homology to jhp0961. These J99-genes are reported to be significantly more frequent in isolates from patients with gastric cancer. Strain B8 also contains a coding sequence HPB8_555 with homology to jhp0950, which is reported to be more frequent in isolates from patients suffering duodenal ulcer [46].

**Figure 5:**
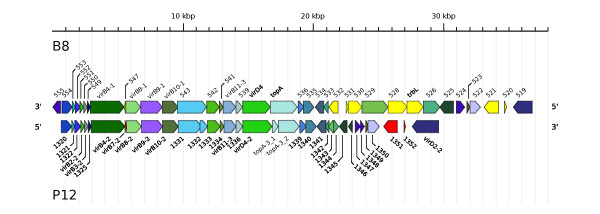
**Comparison of the PZ of strain B8 and the PZ3 of strain P12**. For strain B8, the genomic region from position 490,731 to 527,504 (3'-PZ) is shown. For strain P12, the genomic region from position 1,394,833 to 1,423,818 (PZ3) is shown. Genes with significant similarity (according to blastp hits) are drawn in the same color. Genes with no similarity in the other genome are drawn in solid yellow (B8) and solid red (P12). The image was created by the software AnnotationSketch [67].

In the PZ of strain B8 there are 36 singletons (with respect to the reference strains J99, 26695, HPAG1, and P12) of which 6 are functionally annotated (Additional file 1, Figure S2). Removing P12 from the list of reference strains, one obtains 57 singletons in the PZ of strain B8. Of these singletons, 11 are functionally annotated. The divergent number of singletons inside the PZ of strain B8 can be explained by the fact that the 3'-part of the PZ is highly similar to the plasticity zone PZ3 of strain P12 (personal communication W. Fischer; Figure 5). All coding sequences of the PZ3 of strain P12 are present in the PZ of strain B8, except for the coding sequences *vir*B7-2 and the genes HPP12_1331 and HPP12_1332 of strain P12. The latter two are part of the merged gene HPB8_543.

### Description of the cag pathogenicity island of strain B8

In the genome of strain B8, the *cag *pathogenicity island (*cag*-PAI) is located between position 670,637 and 720,370 (Figure 1). All essential genes for the type IV-secretion system are present in strain B8.

**Figure 6:**
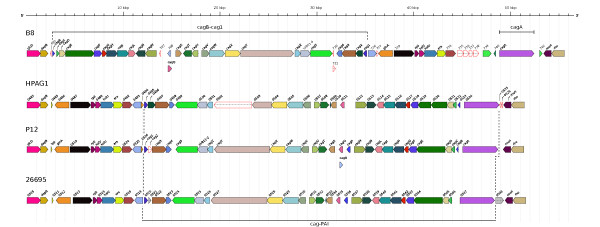
**Similarity comparison of the *cag*-PAI regions of strains B8, HPAG1, P12, and 26695**. The image shows the annotation of genes inside the *cag*-pathogenicity island of the four *H. pylori *genomes. Orthologous groups of genes are drawn in the same color. Each gene is labeled with its 'gene' feature tag from the Genbank annotation file. If for a given gene, a 'gene' feature tag is missing in the Genbank file in the first place, the terminal four characters of the 'locus-tag' feature tag are used as labels instead. Genes which do not show significant sequence similarities to any genes in the other genomes (singletons) are drawn with no fill color but a red stroke color. The image was created by the software AnnotationSketch [67].

**Figure 7:**
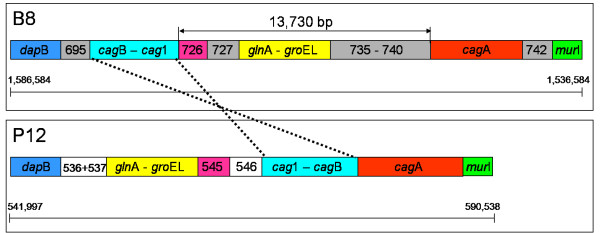
**Similarity comparison of the *cag*-PAI regions of strain B8 and strain P12, restricted to the region between the *dap*B-gene and the *mur*I-gene**. Clusters of genes with homology are shown in the same color. Clusters of genes with no similarity in the other genome are drawn in grey (B8) and white (P12). In strain B8, the region delimited by the *cag*1-gene and the *cag*B-gene is inverted and located 13,730 bp upstream of the *cag*A-gene. A cluster of six genes (four singletons, two variable genes) is located between the *gro*EL-gene and the *cag*A-gene.

Comparing the *cag*-PAI of strain B8 with other *H. pylori*-strains one observes a rearrangement in the region delimited by the *dap*B-gene and the *mur*I-gene, as well as a translocation of the *cag*A-gene. In the *H. pylori *strains 26695, J99, HPAG1, and P12 the regions delimited by the *cag*A-gene and the *cag*1-gene (*cag*-PAI) is located coherently between the *era*-gene (encoding a GTP-binding protein) and the *mur*I-gene (encoding a glutamate racemase). In strain B8 the *cag*A-gene is located separately from the region delimited by the *cag*1-gene and the *cag*B-gene between a cluster of six coding sequences (from HPB8_735 to HPB8_740) upstream of *cag*A on one side and a hypothetical protein next to the *mur*I-gene downstream of the *cag*A-gene on the other side (Figure 6). The coding sequences from HPB8_735 to HPB8_738 are all singletons, while HPB8_739 is a variable gene. With respect to the reference strains, the region delimited by the *cag*1-gene and the *cag*B-gene is inverted and occurs 13,730 bp upstream of the *cag*A-gene (Figure 7). A blastn comparison of different IS-elements of the previously sequenced *H. pylori *genomes against strain B8 gives a hit with bit score 90 to the insertion sequence IS606 (accession number NP_223544) located between the *mur*I-gene and the *cag*A-gene.

The amino acid sequences of the CagA proteins of strain B8 and the reference strains occur highly conserved (88.5% identity). Comparing the amino acid sequences of the CagA EPIYA regions, one observes a much smaller identity of 45.2%. The CagA protein of strain B8 contains the EPIYA motifs A and C which is identical to that of strain 26695. Strain P12 possesses the most pronounced EPIYA motifs (ABCC). Except for strain P12, all strains lack the EPIYA motif B due to a mutation of alanine to threonine resulting in EPIYT.

### Characterization of unknown genes by codon usage analysis

The four singletons HPB8_735, HPB8_736, HPB8_737, and HPB8_738 located within the region separating *cag*A from the gene cluster *cag*B to *cag*1 (Figure 7) are of unknown function and do not show any homology to previously sequenced *H. pylori *strains. The variable gene HPB8_739 is annotated as a regulator of nonsense transcripts. To characterize the origin of the coding sequences from HPB8_735 to HPB8_739, a codon usage analysis was performed (Additional file 1, Figure S5). In particular, the five coding sequences were compared to (a) the *cag*-PAI, (b) to all other coding sequences of strain B8, and (c) to 10 randomly selected coding sequences of strain B8. Additionally, the codon usage of strain B8 was compared to the codon usage of *Helicobacter acinonychis *Sheeba (accession number NC_008229) and *E. coli *K12 (accession number NC_000913), for taking into account the differences of the bacterial genera, see Additional file 1, Figure S5.

Compared to the other genes of strain B8, the group of five genes from HPB8_735 to HPB8_739 as well as the *cag*-PAI show a different codon usage. For the *cag*-PAI, this difference is statistically significant (ANOVA, *p *< 0.01). In contrast, the ten coding sequences randomly selected from strain B8 show a codon usage similar to all other coding sequences of strain B8. The codon usage of strain B8 and *Helicobacter acinonychis *Sheeba is highly similar, whereas the codon usage of *E. coli *K12 is significantly different compared to these two *Helicobacter *strains (ANOVA, *p *< 0.01). This fact suggests the hypothesis that strain B8 acquired the five genes from HPB8_735 to HPB8_739 via horizontal gene transfer from other bacterial species.

### Characterization of the plasmid pHPB8

**Figure 8:**
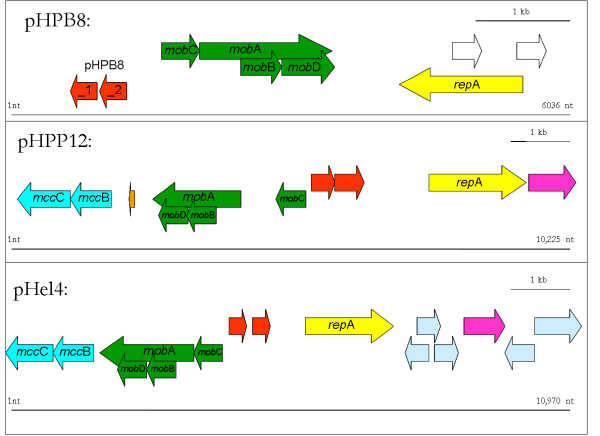
**Functional alignment of the three plasmids of the *H. pylori *strains B8 (pHPB8), P12 (pHPP12), and HPP8 (pHel4)**. Homologuous genes are shown in the same color. The cluster of *mob*-genes (green) and the *rep*A-gene (yellow) are present in all three plasmids. Genes homologuous to pHPB8_1 (plasmid stabilization system protein) and to pHPB8_2 (conserved hypothetical protein) are present in pHPP12 and pHel4 (red). The microcins *mcc*C and *mcc*B are not present in pHPB8. Genes that are not present in the other plasmids are colored white (pHPB8), orange (pHPP12) and light blue (pHel4).

The plasmid pHPB8 of strain B8 has 6,032 bp and a GC content of 35.9%. It contains nine coding sequences (minimum length 80 bp), five of which are functionally annotated (Figure 8). One coding sequence of strain B8 (pHPB8_9) shows homology to the replication initiation protein A (RepA). Furthermore, there are homologies to the *mob*A-gene (pHPB8_4), to the *mob*B-gene (pHPB8_5), to the *mob*C-gene (pHPB8_3), and to the *mob*D-gene (pHPB8_6). The *mob*-genes are reported to encode conjugal mobilization proteins. In pHPB8 they are organized in a cluster. pHPB8 was compared to pHPP12 (the plasmid of strain P12, 10,225 bp, 11 coding sequences) and pHel4 (the plasmid of strain P8, 10,970 bp, 15 coding sequences [47]). In all three plasmids the *rep*A-gene and the cluster of *mob*-genes are present. Additionally, all three plasmids contain a coding sequence for a plasmid stabilization system protein (pHPB8_1) and a conserved hypothetical protein (pHPB8_2). The *mcc*C-gene and *mcc*B-gene coding for microcin are present in pHel4 and pHPP12, but not in pHPB8.

## Discussion

The Gram-negative pathogen *H. pylori *is an interesting model system for microorganisms persisting in the host for decades. To study the adaptation and persistence process in the stomach, an animal model mimicking the human situation is required. The Mongolian gerbil model is a suitable model, as it was shown that a lasting *H. pylori*-infection results in the gastric carcinogenic pathway [48] via gastritis, atrophy, metaplasia, and dysplasia, and finally inducing gastric adenocarcinoma [11,36,37]. These gerbils were infected with classified *H. pylori *type I-strains, expressing a functional T4SS able to translocate the oncoprotein CagA into the host cells, where it can be tyrosin-phosphorylated by host kinases [8,49,50]. In a time course study the gerbils were challenged with a gerbil-adapted *H. pylori*-strain B8, originating from the human isolate B128. To improve its adaptation, strain B8 was passaged several times through stomach of Mongolian gerbils from our breeding colony.

Up to now there are nine finished whole genome sequences of different *H. pylori*-strains available in DDBJ/EMBL/Genbank [27,28,30-34]. All these strains are human isolates representing genetic features of specific gastroduodenal diseases, such as gastritis, peptic ulcer, and malignant sequelae. For a better understanding of the *H. pylori*-induced gastric pathogenesis and its basic molecular mechanism involved, the complete sequencing of the pathogen is a good approach.

For the current study we sequenced, annotated, and analyzed for the first time the whole genome of a gerbil-adapted *H. pylori*-strain. One goal of this study was to elucidate the effect of adaptation of the parental strain B128 on the genome level. Since the genome of strain B128 consists of 73 supercontigs (thus it is not fully sequenced yet), another goal was the comparative analysis to other available fully sequenced *H. pylori *genomes.

The genome comparison of strain B8 with the recently sequenced parental strain B128 reveals that all 73 supercontigs can be mapped to the finished genome of strain B8 covering about 98% of the sequence. The uncovered genome regions of strain B8 contain 60 coding sequences (partly or completely). For 42 of these coding sequences there is no 80/80 blastn hit somewhere in the genome of strain B128. Therefore, in a strict sense these coding sequences are not strain-specific, because they are likely to occur in a completely sequenced genome of strain B128 (whose gaps would be closed). Furthermore, 1,281 genes of strain B8 (74.9%) completely match with no differences to the B128-supercontigs, and 1,652 genes of strain B8 (96.6%) completely match with less than 2% differences (i.e. insertions, deletions, and replacements). This reveals that the supercontigs of strain B128 are highly identical to the finished sequence of strain B8. About 20% of the coding sequences of strain B8 have between one and 40 differences, whereas only 1.5% of these coding sequences were found with two or more differences. At this point it is not possible to elucidate exactly the cause of these observations. Some of these differences may be due to the stomach passages, others due to sequencing errors.

In total, we found 52 genes of strain B8 (i.e. 3%) with a length variation when comparing all genes of strain B8 with the other *H. pylori *strains (data not shown). Eppinger *et al*. [51] found 92 fragmented genes in *H. acinonychis *representing a ratio of 6%. This higher ratio may be due to a host jump of *H. acinonychis *from human to large felines. However, we have to consider that the adaptation from early humans to large felines is a much longer process (thousands of years) than the adaptation of the parental strain B128 to the Mongolian gerbil during several stomach passages.

Resequencing the fragmented genes by Sanger technology, we were able to eliminate a homopolymer error in the 454-reads [52]. In only two cases we had to revise the sequence, all other fragmented or length variable genes were confirmed. These results indicate that the combination of 454-pyrosequencing (with high coverage) and Sanger-sequencing (with low coverage) delivered a high quality sequence, which, before gap closure, consisted of only 29 supercontigs.

Comparing the predicted proteins of strains B8 and B128, one obtains 1,711 - 673 = 1,038 proteins of strain B8 that have a 100/100 blastp hit in strain B128 (Additional file 1, Table S4, first row). When using the less stringent 80/80 blastp hit criterion for the comparison, there are 425 predicted proteins in strain B8 and 371 (87%) of the corresponding coding sequences match completely with at most 2% differences to the genome of strain B128. This indicates that in most cases the DNA sequence is present in strain B128, but the corresponding coding sequence has not been annotated sufficiently. Out of the remaining 54 singletons, 42 have to be regarded as "weak" (possible) and 12 as "strong" (definitive) singletons, of which 5 are functionally annotated (Table 4). Interestingly the genes HPB8_138 (*ton*B) and HPB8_888 (*feo*B) are both reported to have an important role in iron acquisition. The iron repressible outer membrane protein TonB possibly serves as a receptor for the uptake of heme [53], whereas FeoB is reported to act as a high affinity Fe^2+ ^transporter [54]. The gene HPB8_692 (*pgb*A) encodes a plasminogen-binding protein. In previous studies it was demonstrated, that PgbA intervenes with the mammalian proteolytic plasminogen-plasmin system [55]. Due to the fact that interaction with the plasminogen system promotes damage of extracellular matrices and bacterial spread, plasminogen binding activity might be relevant for pathogenesis [56]. Further genes are related to chemotaxis (HPB8_1483) and hydrogen metabolism (HPB8_655). Possibly these genes are important for the adaptation process during the stomach passages in the Mongolian gerbils. HPB8_138 (*ton*B), HPB8_692 (*pgb*A), and HPB8_655 (*hyp*D2) are of special interest, because they also appear as singletons when comparing strain B8 against the reference strains 26695, J99, P12, and HPAG1. We remark that our definition of singletons is based on comparisons of the genes on the protein sequence level. Thus the singletons may include highly variable genes (e.g. *ton*B).

Using even less stringent parameters (blastp match of bit score at least 100) 182 strain-specific genes in strain B8 are obtained (data not shown). In contrast to the singletons (most of them are hypothetical proteins), the genes with length variations (as mentioned above) may also be candidates for explaining the gerbil-adaptation. These genes need to be studied further to understand the adaptation mechanism to the gerbil gastric mucosa.

The gene annotation for the genome of strain B128 was done automatically. Unfortunately, there is no functional annotation to any of the coding sequences: all coding sequences are annotated as 'hypothetical protein'. However, functional annotations or further homology information can be derived for many genes of strain B128 by exploiting the fact that there are 1,269 pairs of orthologous genes between strain B128 and B8. Among these, there are 1,169 genes in strain B8 that have functional annotations, or for which homology to genes in other genomes exist. Each of these genes suggests a reasonable annotation for the coding sequences of strain B128 which in turn would allow to considerably improve the annotation of the genome of strain B128. Of course, a final conclusive comparative analysis of the genome of strain B8 versus the parental strain B128 would require to close the gaps of strain B128 and to improve the annotation.

The genome of strain B8 consists of a single circular chromosome of 1,673,997 bp with a GC content of 38.8%. It contains 1,711 coding sequences (average length 897 bp), 54.3% of which are functionally annotated. The general features of the gerbil-adapted strain B8 are consistent with other four sequenced genomes (strains 26695 [27], J99 [28], HPAG1 [30], P12), except that strain B8 has more coding sequences (7.9% more than strain 26695, 13% more than strain J99, 10.2% more than strain HPAG1 and 8.4% more than strain P12) and considerably more strain-specific coding sequences. In particular, there are 44% more strain-specific genes in strain B8 than in any of the other analyzed *H. pylori *genomes. This is supported by the large number of phase variable genes, building a genetic pool for possible adaptation processes.

Among the 293 strain-specific coding sequences there are several DNA methylases, restriction endonucleases, and DNA transfer proteins supporting the genetic diversification process of *H. pylori*. The analysis of the singletons of strain B8 according to the pathogenicity of the reference strains revealed a remarkable difference in the VirB and VirD proteins of the additional T4SS (*tfs*3) between the duodenal ulcer and gastritis strains. Nevertheless, no clear tendency could be demonstrated for the pathogenicity groups since Israel *et al*. presented a strain (G1.1) isolated from a duodenal ulcer patient that did not carry a functional T4SS [12]. Thus, a functional T4SS might not be necessary for developing duodenal ulcer. Interestingly three DNA modification genes (HPB8_537, HPB8_1098, HPB8_1516) were also present in the microarray study of the peptic ulcer strains J99 and B128 [12]. This suggests the hypothesis that these genes may be involved in the development of gastroduodenal lesions.

Strain B8 contains a 6,032 bp plasmid (pHPB8) with a GC content of 35.9%. The plasmid has nine coding sequences, five of which are functionally annotated. pHPB8 is on of the smallest *H. pylori *plasmids isolated so far, but nevertheless it encodes the expected replication initiation protein A (RepA) and the cluster of four conjugal mobilization proteins (Mob) as well as a plasmid stabilization system protein. Our comparative analysis with the B128-supercontigs indicates that the parental strain B128 already contains this strain-specific plasmid pHPB8. However, this was neither annotated as such, nor mentioned in the publication of McClain *et al*. [35].

A genome comparison of strain B8 versus strains 26695 and J99 based on 80/80 blastn hits and visualized by the Artemis Comparison Tool (ACT) [57] reveals a large PZ of 81 kbp, containing 84 coding sequences with a GC content of 34%. The 3'-region of the PZ of strain B8 is very similar to the PZ3 of strain P12 (29 kbp). It is shown that this PZ3 belongs to a type 2 TnPZ, encoding a novel T4SS-3 (*tfs*3) flanked by direct repeats of 5'-AAGAATG-3' [42]. Most coding sequences of the *tfs*3 of strain P12 (Table 1) have a corresponding coding sequence in the T4SS of strain B8. The *tfs*3 of strain B8 has one coding sequence less (HPP12_*vir*B-2) and a merged coding sequence HPB8_543 of which the first part corresponds to the coding sequence HPP12_1331 and the second part to the coding sequence HPP12_1332 (Figure 5). Besides the *tfs*3, there are several typical coding sequences in the PZ of strain B8: a flanking 5S/23S-rRNA gene pair, the *top*A-gene (DNA topoisomerase I), the *vir*D2-gene, the *par*A-gene (putative chromosome partitioning protein), the *orf*Q-gene (DNA methylase and helicase), the *xer*D-gene (integrase/recombinase), and a transposable element IS608. Moreover, several of the singletons of strain B8 are located within its PZ. Interestingly, the PZ of strain B8 contains several coding sequences (HPB8_514, HPB8_512, HPB8_506, HPB8_474, HPB8_473, and HPB8_555) that show homology to genes reported to be significantly more frequent in isolates of patients suffering from gastroduodenal diseases such as peptic ulcer and gastric cancer [46].

The more virulent *H. pylori *type I-strains are expressing a functional T4SS that is encoded on the *cag*-PAI. The gerbil-adapted type I-strain B8 was used to study the role of the *cag*-PAI on the development of precancerous conditions in Mongolian gerbils [36,37]. PCR-amplification of the *cag*A-gene starting from adjacent genes, using *H. pylori *26695 as reference sequence, did not lead to an amplification product. This discrepancy can be explained by the fact that the *cag*-PAI of strain B8 has a rearrangement between the *dap*B-gene and the *mur*I-gene. Moreover, there is a translocation of the *cag*A-gene 13,730 bp downstream of the inverted gene cluster from the *cag*B-gene to the *cag*1-gene. Interestingly, there are four hypothetical proteins and one variable gene directly adjacent to *cag*A. To derive hypothesis of its origin, a codon usage analysis was performed. This involves the *cag*-PAI genes, the five coding sequences from HPB8_735 to HPB8_739 and ten randomly selected genes of strain B8, as well as all remaining genes of strain B8. This codon usage of the *cag*-PAI and of the five genes from HPB8_735 to HPB8_739 significantly differs from the codon usage of the other two groups of coding sequences. This suggests that strain B8 acquired the five coding sequences with unknown function and possibly also the *cag*-PAI via horizontal gene transfer.

## Conclusion

In this current study we sequenced and annotated the whole genome of the gerbil-adapted *H. pylori*-strain B8 (accession numbers: FN598874 for the genome, FN665651 for the plasmid). The genome analysis suggests that this type I-strain possibly has acquired the virulence mechanism encoded in the *cag*-PAI as well as other adjacent unknown genes via horizontal gene transfer. This may have occurred during microevolution optimizing the adaptation to its hostile niche, the gastric mucosa. The relatively large number of singletons, the existence of length variable genes, and the large PZ may already reflect an adaptation-process to the gerbil stomach. Altogether, this pathogen may use its dynamic pool of genetic variants, representing a sufficient genetic diversity to allow *H. pylori *to occupy all of the potential niches in the stomach.

## Methods

### Bacterial strain

*H. pylori *B128 was isolated from a human gastric ulcer patient and afterward subsequently passaged through gerbil stomachs until adaptation. In our hands, after several further stomach passages of up to four weeks, this strain was adapted to our in-house Mongolian gerbil out-bred line. Furthermore, a streptomycin resistance was introduced for a successful quantitative reisolation. For an unmistakable differentiation we named our gerbil-adapted *H. pylori*-strain B8. This strain was used for the whole genome sequencing project described in this manuscript.

All animal experiments and procedures carried out were conducted in accordance with the Guidelines for the Care and Use of Laboratory Animals and approved by the Regierung von Oberbayern (AZ 55.2-1-54-2531-41/04 and 55.2-1-54-2531-78/05).

### Genome sequencing, assembly and gap closure

A combination of Sanger sequencing and pyrosequencing technologies was used for whole-genome sequencing of *Helicobacter pylori *strain B8. Total genomic DNA of a liquid *H. pylori *B8 culture (Brucella broth, 10% FCS, streptomycin 250 mg/l) was extracted by using a genomic-tip G-500 (Qiagen, Hilden, Germany). To construct plasmid libraries for Sanger sequencing, the DNA was sheared by employing a Hydroshear as described by the manufacturer (GeneMachines, San Carlos, CA, USA). The resulting DNA fragments were separated by gel electrophoresis. Fragments of 1.5 to 3.0 kbp were isolated and cloned into the vector pCR4.1-TOPO by employing the TOPO-TA Cloning Kit for Sequencing (Invitrogen, Karlsruhe, Germany). Subsequently, recombinant plasmids were automatically isolated by using a BioRobot 8000 (Qiagen GmbH, Hilden, Germany). The insert ends of 5285 recombinant plasmids were sequenced by using dye terminator chemistry and an ABI Prism 3730XL DNA sequencer (Applied Biosystems, Foster City, CA, USA). The resulting sequences were processed with the Phred program and assembled into contigs by using the Phrap assembly tool [58]. The genomic DNA of *H. pylori *B8 was also sequenced by conducting runs (70 × 75 picotitre plates) on a Roche GS-FLX pyrosequencer (Roche, Mannheim, Germany). The preparation of DNA and pyrosequencing was done according to the manufacturer's protocols (Roche). The sequenced 167,448 pyrosequencing reads were assembled into 50 contigs *>*500 bp using the Newbler Assembler (Roche). Sequence editing of shotgun sequences and pyrosequences was performed by using the GAP4 program of the Staden software package [59]. In summary, a 16-fold coverage was obtained after assembly of the pyrosequencing-derived sequences and a 2.5-fold coverage by using Sanger reads only. To solve misassembled regions and to close the remaining 29 gaps in the genomic sequence, PCR and combinatorial multiplex PCR on isolated genomic DNA as well as primer walking on recombinant plasmids were performed. PCR reactions were carried out with the 5-Prime Extender Polymerase System as described by the manufacturer. In addition, the TempliPhi™ Sequence Resolver Kit was used for the sequencing of problematic templates, i.e., templates harboring stable secondary structures (Illustra™ TempliPhi™ Sequence Resolver Kit, GE Healthcare).

### Annotation and Comparative Genome Analysis

The complete genome sequence of *H. pylori *was automatically annotated using the GenDB [60] genome annotation system. This applies a combined gene prediction strategy based on GLIMMER 2.1 and CRITICA, along with post-processing by RBSfinder. Subsequently, for all predicted proteins searches in public databases, including SWISS-PROT, TrEMBL, Pfam, KEGG, and COG were performed. The InterPro database was used to infer GO numbers. Additional observations about the predicted proteins were obtained by applying the programs helix-turn-helix, TMHMM, and SignalP. All observations delivered by the different searches were manually inspected to infer functional annotations for the predicted proteins. In case of doubt, additional blast searches were performed. The genome of strain B8 was deposited in DDBJ/EMBL/Genbank on December 1, 2009 and has accession number FN598874. The plasmid of strain B8 was deposited in DDBJ/EMBL/Genbank on January 26, 2010 and has accession number FN665651.

The EDGAR-software [40] was used to compare the proteomes of five completely sequenced *H. pylori *strains and to identify common, unique, and orthologous genes. A gene whose description does not contain the keywords *hypothetical *or the keyword *putative *is considered a gene with known function. We also use the notion *functionally annotated*.

The set of genes of a reference strain for which an orthologous gene can be identified in every other strain is referred to as the core genome. In contrast, genes of the reference strain with no ortholog in any other strain are called singletons or strain-specific.

### Mapping of the B128-supercontigs to strain B8

The genome sequence of strain B128 is available in DDBJ/EMBL/Genbank under the project accession number ABSY00000000. The 73 Genbank formatted files in this project (accession numbers ABSY01000001-ABSY01000073) were downloaded from Genbank on June 12, 2009. Each file gives the sequence and annotation of an assembled contig. The supercontigs are sorted in descending order of their size which ranges from 226,574 bp (for supercontig ABSY01000001) down to 649 bp (for supercontig ABSY01000073).

The genome sequences of strain B128 was extracted from the genbank files and matched against the complete genome of strain B8 using the Nucmer program from the MUMmer software suite [61]. More precisely, Nucmer computed maximal matches of minimum length 18. The resulting .coords-file was read by the program OSLay [62]. This delivered an optimal syntenic layout of 71 B128-supercontigs relative to the genome sequence of strain B8. The remaining two supercontigs (supercontig 146 and supercontig 161) were mapped to the plasmid of strain B8, according to high scoring blastn hits. The resulting mapping thus assigns to each B128-supercontig a unique region of the genome of strain B8. Close inspection of the mapping shows that there are five regions where B128-supercontigs pairwise overlap each other by at least 73 bases. This suggests that these B128-supercontigs could have been assembled to larger supercontigs.

The quality of the mapping was verified by matching the B128-supercontigs to the assigned regions of strain B8 using Vmatch [63]. When restricting to matches with at least 90% sequence identity, on average 99.8% of the lengths of the B128-supercontigs map to the assigned regions of strain B8 at an average sequence identity of 99.7%. These numbers show that both genomes are highly similar.

While the B128-supercontigs are contained in the genome of strain B8, the latter has additional sequence content relative to strain B128, namely the sequence uncovered by the B128-supercontigs. We refer to them as uncovered regions of HBP8. There are 63 uncovered regions whose length ranges from 1 to 4,608 bp. The total length of the uncovered regions is 35,157 bp (average length 558 bp). This is 2% of the entire genome sequence of strain B8. There are 20 coding sequences of strain B8 which are fully contained in uncovered regions and 33 coding sequence of which parts are in uncovered regions.

### Repeat Analysis

Here we consider repeats as regions in a genome that are duplicated and highly similar. The program Vmatch [63] was used to compute repeats in the chromosomes of the different *H. pylori *strains. We were in particular interested in repeats of length at least 100, such that the two instances of the repeat have sequence identity of at least 80%. This identity threshold is consistent with the threshold used in the repeat counting method of [64].

### Plasticity Zones

Plasticity zones of strain 26695 and J99 were mainly derived from [42], but also from [28]. The positions of the genes mentioned in these papers were taken from the corresponding Genbank-entries. In strain J99 the PZ is enveloped by one of the 23S:5S genes and the *fts*Z gene. These genes were treated as the first genes not belonging to the PZ. The same was done for the genome of strain 26695, with the difficulty that the PZ is divided into two regions: the last genes before the left PZ region are 23S:5S and the first gene after the right PZ region is *fts*Z (nomenclature from [42]). PZs for strain P12 were inferred from a visualization delivered by the Artemis Comparison Tool (ACT) [57], when comparing strain P12 with strain HPAG1. The PZ for strain B8 was found using ACT. It was most obvious when comparing it to HPAG1.

### Analysis of the codon usage

The codon usage analysis was done with the program codonw [65]. ANOVA was done for each amino acid using R [66]. As the the data did not show a normal distribution and the sample sizes were very different, a smaller *p*-value threshold of *p *< 0.01 was used, instead of the standard threshold of *p *< 0.05. To identify the differences, the Tuckey HSD (honestly significant difference) test was used, again with a 99% confidence-level. This test allows to identify which means contribute to the overall significance found with the ANOVA.

### *H. pylori* reference genomes

The following reference genomes proved valuable in refining the automatic annotation of GenDB: **26695 **Genbank entry (accession number NC_000915) from 29-NOV-2007; **J99 **Genbank entry (accession number NC_000921) from 29-NOV-2007; **HPAG1 **Genbank entry (accession number NC_008086) from 07-DEC-2007 **P12 **Genbank entry (accession number NC_011498) from 28-APR-2009; **Shi470 **Genbank entry (accession number NC_010698) from 17-MAY-2008

## Authors' contributions

MF closed the gaps and performed various sequence analyses. TJ annotated the genome and performed various bioinformatics analyses. DW performed the codon usage analysis and applied several bioinformatics tools. RD supervised the sequencing, RH conceived the project, AG conducted the automatic annotation with GenDB, SK supervised the bioinformatics analysis, and GR conceived the study. GR, SK, and MF wrote the paper. All authors read and approved the final manuscript.

## Supplementary Material

Additional file 1 **This file contains 11 tables and 7 figures with additional information**. Here is a list of abbreviations of the table and figure captions with the page numbers in Additional file 1 where they start:
         Supplementary Tables   
   S1 Regions of strain B8 covered by supercontigs of strain B128 1   
   S2 Pairs of B128-supercontigs with overlaps 4   
   S3 List of genes in uncovered regions of strain B8 5   
   S4 Distribution of the number of B8 proteins with no hit in strain B128 7   
   S5 List of strong singletons 8   
   S6 List of weak singletons 18   
   S7 List of singletons of strain B128 vs. strain B8 20   
   S8 List of singletons of strain B8 vs. four reference strains 21   
   S9 List of singletons of strain B8 versus strains J99 and P12 29   
   S10 List of singletons of strain B8 versus strains 26695 and HPAG1 30   
   S11 List of coding sequences in the plasticity zone of strain B8 32   
         Supplementary Figures   
   S1 Distribution of the repeat density for 1053 bacterial genomes 35   
   S2 Comparison of genes in the plasticity zones of four *H. pylori *strains 36   
   S3 Synteny plot of strain B8 vs. strain J99 37   
   S4 Synteny plot of strain B8 vs. strain P12 37   
   S5 Distribution of relative codon usage 39   
   S6 Codon usage of four groups of B8 genes 40   
   S7 Codon usage of strain B8, *H. acinonychis *Sheeba, and *E. coli *K12 41    Click here for file

## References

[B1] Suerbaum S Michetti P *Helicobacter pylori *infection N Engl J Med 2002 347 1175 1186 10.1056/NEJMra020542 12374879

[B2] International Agency for Research on Cancer Schistosomes, liver flukes and *Helicobacter pylori*. IARC Working Group on the Evaluation of Carcinogenic Risks to Humans. Lyon, 7-14 June 1994 IARC Monogr Eval Carcinog Risks Hum 1994 61 1 241 7715068 PMC7681621

[B3] Rad R Dossumbekova A Neu B Lang R Bauer S Saur D Gerhard M Prinz C Cytokine gene polymorphisms influence mucosal cytokine expression, gastric inflammation, and host specific colonisation during *Helicobacter pylori *infection Gut 2004 53 8 1082 9 10.1136/gut.2003.029736 15247172 PMC1774164

[B4] Ogihara A Kikuchi S Hasegawa A Kurosawa M Miki K Kaneko E Mizukoshi H Relationship between *Helicobacter pylori *infection and smoking and drinking habits J Gastroenterol Hepatol 2000 15 3 271 6 10.1046/j.1440-1746.2000.02077.x 10764027

[B5] Cover TL Blanke SR *Helicobacter pylori *VacA, a paradigm for toxin multifunctionality Nat Rev Microbiol 2005 3 320 332 10.1038/nrmicro1095 15759043

[B6] Sewald X Gebert-Vogl B Prassl S Barwig I Weiss E Fabbri M Osicka R Schiemann M Busch D Semmrich M Holzmann B Sebo P Haas R Integrin subunit CD18 is the T-lymphocyte receptor for the *Helicobacter pylori *vacuolating cytotoxin Cell Host Microbe 2008 3 20 9 10.1016/j.chom.2007.11.003 18191791

[B7] Miura M Ohnishi N Tanaka S Yanagiya K Hatakeyama M Differential oncogenic potential of geographically distinct *Helicobacter pylori cag*A isoforms in mice Int J Cancer 2009 125 11 2497 504 10.1002/ijc.24740 19588494

[B8] Hatakeyama M *Helicobacter pylori *and gastric carcinogenesis J Gastroenterol 2009 44 4 239 48 10.1007/s00535-009-0014-1 19271114

[B9] Backert S Selbach M Role of type IV secretion in *Helicobacter pylori *pathogenesis Cell Microbiol 2008 10 1573 1581 10.1111/j.1462-5822.2008.01156.x 18410539

[B10] Philpott D Belaid D Troubadour P Thiberge J Tankovic J Labigne A Ferrero R Reduced activation of inflammatory responses in host cells by mouse-adapted *Helicobacter pylori *isolates Cell Microbiol 2002 4 5 285 96 10.1046/j.1462-5822.2002.00189.x 12064285

[B11] Watanabe T Tada M Nagai H Sasaki S Nakao M *Helicobacter pylori *infection induces gastric cancer in Mongolian gerbils Gastroenterol 1998 115 3 642 8 10.1016/S0016-5085(98)70143-X 9721161

[B12] Israel DA Salama N Arnold CN Moss SF Ando T Wirth HP Tham KT Camorlinga M Blaser MJ Falkow S Peek RM *Helicobacter pylori *strain-specific differences in genetic content, identified by microarray, influence host inflammatory responses J Clin Invest 2001 107 611 620 10.1172/JCI11450 11238562 PMC199426

[B13] Franco AT Israel DA Washington MK Krishna U Fox JG Rogers AB Neish AS Collier-Hyams L Perez-Perez GI Hatakeyama M Whitehead R Gaus K O'Brien DP Romero-Gallo J Peek RM Activation of β-catenin by carcinogenic *Helicobacter pylori* Proc Natl Acad Sci USA 2005 102 10646 10651 10.1073/pnas.0504927102 16027366 PMC1180811

[B14] Correa P A human model of gastric carcinogenesis Cancer Res 1988 48 13 3554 60 3288329

[B15] Kang J Blaser M Bacterial populations as perfect gases: genomic integrity and diversification tensions in *Helicobacter pylori* Nat Rev Microbiol 2006 4 11 826 36 10.1038/nrmicro1528 17041630

[B16] Suerbaum S Achtman M Evolution of *Helicobacter pylori*: the role of recombination Trends Microbiol 1999 7 5 182 4 10.1016/S0966-842X(99)01505-X 10383222

[B17] Wang G Humayun M Taylor D Mutation as an origin of genetic variability in *Helicobacter pylori* Trends Microbiol 1999 7 12 488 93 10.1016/S0966-842X(99)01632-7 10603484

[B18] Weiser J Love J Moxon E The molecular mechanism of phase variation of *H. influenzae *lipopolysaccharide Cell 1989 59 4 657 65 10.1016/0092-8674(89)90011-1 2479481

[B19] Aras RA Kang J Tschumi AI Harasaki Y Blaser MJ Extensive repetitive DNA facilitates prokaryotic genome plasticity Proc Natl Acad Sci USA 2003 100 13579 13584 10.1073/pnas.1735481100 14593200 PMC263856

[B20] Salaün L Linz B Suerbaum S Saunders NJ The diversity within an expanded and redefined repertoire of phase-variable genes in *Helicobacter pylori* Microbiology 2004 150 817 830 10.1099/mic.0.26993-0 15073292

[B21] Israel D Lou A Blaser M Characteristics of *Helicobacter pylori *natural transformation FEMS Microbiol Letters 2000 186 2 275 80 10.1111/j.1574-6968.2000.tb09117.x 10802184

[B22] Suerbaum S Smith J Bapumia K Morelli G Smith N Kunstmann E Dyrek I Achtman M Free recombination within *Helicobacter pylori* Proc Natl Acad Sci USA 1998 95 21 12619 24 10.1073/pnas.95.21.12619 9770535 PMC22880

[B23] Achtman M Azuma T Berg D Ito Y Morelli G Pan Z Suerbaum S Thompson S van der Ende A van Doorn L Recombination and clonal groupings within *Helicobacter pylori *from different geographical regions Mol Microbiol 1999 32 3 459 70 10.1046/j.1365-2958.1999.01382.x 10320570

[B24] Falush D Wirth T Linz B Pritchard J Stephens M Kidd M Blaser M Graham D Vacher S Perez-Perez G Yamaoka Y Mégraud F Otto K Reichard U Katzowitsch E Wang X Achtman M Suerbaum S Traces of human migrations in *Helicobacter pylori *populations Science 2003 299 5612 1582 5 10.1126/science.1080857 12624269

[B25] Linz B Balloux F Moodley Y Manica A Liu H Roumagnac P Falush D Stamer C Prugnolle F van der Merwe S Yamaoka Y Graham D Perez-Trallero E Wadstrom T Suerbaum S Achtman M An African origin for the intimate association between humans and *Helicobacter pylori* Nature 2007 445 7130 915 8 10.1038/nature05562 17287725 PMC1847463

[B26] Gressmann H Linz B Ghai R Pleissner K Schlapbach R Yamaoka Y Kraft C Suerbaum S Meyer T Achtman M Gain and loss of multiple genes during the evolution of *Helicobacter pylori* PLoS Genetics 2005 1 4 e43 10.1371/journal.pgen.0010043 16217547 PMC1245399

[B27] Tomb J White O Kerlavage A Clayton R Sutton G Fleischmann R Ketchum K Klenk H Gill S Dougherty B Nelson K Quackenbush J Zhou L Kirkness E Peterson S Loftus B Richardson D Dodson R Khalak H Glodek A McKenney K Fitzegerald L Lee N Adams M Hickey E Berg D Gocayne J Utterback T Peterson J Kelley J Cotton M Weidman J Fujii C Bowman C Watthey L Wallin E Hayes W Borodovsky M Karp P Smith H Fraser C Venter J The complete genome sequence of the gastric pathogen *Helicobacter pylori* Nature 1997 388 6642 539 47 10.1038/41483 9252185

[B28] Alm R Ling L Moir D King B Brown E Doig P Smith D Noonan B Guild B deJonge B Carmel G Tummino P Caruso A Uria-Nickelsen M Mills D Ives C Gibson R Merberg D Mills S Jiang Q Taylor D Vovis G Trust T Genomic-sequence comparison of two unrelated isolates of the human gastric pathogen *Helicobacter pylori* Nature 1999 397 6715 176 80 10.1038/16495 9923682

[B29] Alm R Trust T Analysis of the genetic diversity of *Helicobacter pylori*: the tale of two genomes J Mol Med 1999 77 12 834 46 10.1007/s001099900067 10682319

[B30] Oh J Kling-Bäckhed H Giannakis M Xu J Fulton R Fulton L Cordum H Wang C Elliott G Edwards J Mardis E Engstrand L Gordon J The complete genome sequence of a chronic atrophic gastritis *Helicobacter pylori *strain: evolution during disease progression Proc Natl Acad Sci USA 2006 103 26 9999 10004 10.1073/pnas.0603784103 16788065 PMC1480403

[B31] Dong Q Wang Q Xin Y Li N Xuan S Comparative genomics of *Helicobacter pylori* World J 2009 15 32 3984 91 10.3748/wjg.15.3984 PMC2731947 19705492

[B32] *Helicobacter pylori *G27 and Related Genome Resources http://hpylori.ucsc.edu/

[B33] Baltrus D Amieva M Covacci A Lowe T Merrell D Ottemann K Stein M Salama N Guillemin K The complete genome sequence of *Helicobacter pylori *strain G27 J Bacteriol 2009 191 447 8 10.1128/JB.01416-08 18952803 PMC2612421

[B34] Giannakis M Chen S Karam S Engstrand L Gordon J *Helicobacter pylori *evolution during progression from chronic atrophic gastritis to gastric cancer and its impact on gastric stem cells Proc Natl Acad Sci USA 2008 105 11 4358 4363 10.1073/pnas.0800668105 18332421 PMC2393758

[B35] McClain MS Shaffer CL Israel DA Peek R Cover TL Genome sequence analysis of *Helicobacter pylori *strains associated with gastric ulceration and gastric cancer BMC Genomics 2009 10 3 10.1186/1471-2164-10-3 19123947 PMC2627912

[B36] Rieder G Merchant J Haas R *Helicobacter pylori cag*-type IV secretion system facilitates corpus colonization to induce precancerous conditions in Mongolian gerbils Gastroenterol 2005 128 5 1229 42 10.1053/j.gastro.2005.02.064 15887107

[B37] Wiedemann T Loell E Mueller S Stoeckelhuber M Stolte M Haas R Rieder G *Helicobacter pylori cag*-Pathogenicity island-dependent early immunological response triggers later precancerous gastric changes in Mongolian gerbils PloS One 2009 4 3 e4754 10.1371/journal.pone.0004754 19270747 PMC2650263

[B38] Shak JR Dick JJ Meinersmann RJ Perez-Perez GI Blaser MJ Repeat-associated plasticity in the *Helicobacter pylori *RD gene family J Bacteriol 2009 191 6900 6910 10.1128/JB.00706-09 19749042 PMC2772487

[B39] Bergman M Del Prete G van Kooyk Y Appelmelk B *Helicobacter pylori *phase variation, immune modulation and gastric autoimmunity Nat Rew Microbiol 2006 4 2 151 9 10.1038/nrmicro1344 16415930

[B40] Blom J Albaum S Doppmeier D Pühler A Vorhölter F Zakrzewski M Goesmann A EDGAR: a software framework for the comparative analysis of prokaryotic genomes BMC Bioinformatics 2009 10 154 10.1186/1471-2105-10-154 19457249 PMC2696450

[B41] Altschul S Madden T Schäffer A Zhang J Zhang Z Miller W Lipman D Gapped BLAST and PSI-BLAST: a new generation of protein database search programs Nucleic Acids Res 1997 25 17 3389 402 10.1093/nar/25.17.3389 9254694 PMC146917

[B42] Kersulyte D Lee W Subramaniam D Anant S Herrera P Cabrera L Balqui J Barabas O Kalia A Gilman R Berg D *Helicobacter pylori *'s plasticity zones are novel transposable elements PloS One 2009 4 9 e6859 10.1371/journal.pone.0006859 19727398 PMC2731543

[B43] de Jonge R Kuipers EJ Langeveld SC Loffeld RJ Stoof J van Vliet AH Kusters JG The *Helicobacter pylori *plasticity region locus jhp0947-jhp0949 is associated with duodenal ulcer disease and interleukin-12 production in monocyte cells FEMS Immunol Med Microbiol 2004 41 161 167 10.1016/j.femsim.2004.03.003 15145461

[B44] Occhialini A Marais A Alm R Garcia F Sierra R Mégraud F Distribution of open reading frames of plasticity region of strain J99 in *Helicobacter pylori *strains isolated from gastric carcinoma and gastritis patients in Costa Rica Infect Immun 2000 68 11 6240 9 10.1128/IAI.68.11.6240-6249.2000 11035731 PMC97705

[B45] Santos A Queiroz D Ménard A Marais A Rocha G Oliveira C Nogueira A Uzeda M Mégraud F New pathogenicity marker found in the plasticity region of the *Helicobacter pylori *genome J Clin Microbiol 2003 41 4 1651 5 10.1128/JCM.41.4.1651-1655.2003 12682156 PMC153914

[B46] Romo-González C Salama NR Burgeõ Ferreira J Ponce-Castañeda V Lazcano-Ponce E Camorlinga-Ponce M Torres J Differences in genome content among *Helicobacter pylori *isolates from patients with gastritis, duodenal ulcer, or gastric cancer reveal novel disease-associated genes Infect Immun 2009 77 2201 2211 10.1128/IAI.01284-08 19237517 PMC2681767

[B47] Hofreuter D Haas R Characterization of two cryptic *Helicobacter pylori *plasmids: a putative source for horizontal gene transfer and gene shuffling J Bacteriol 2002 184 2755 2766 10.1128/JB.184.10.2755-2766.2002 11976306 PMC135030

[B48] Dixon M Genta R Yardley J Correa P Classification and grading of gastritis. The updated Sydney System. International Workshop on the Histopathology of Gastritis, Houston 1994 Am J Surg Pathol 1996 20 10 1161 81 10.1097/00000478-199610000-00001 8827022

[B49] Odenbreit S Kavermann H Püls J Haas R *Cag*A tyrosine phosphorylation and interleukin-8 induction by *Helicobacter pylori *are independent from *alp*AB, HopZ and bab group outer membrane proteins Int J Med Microbiol 2002 292 3-4 257 66 10.1078/1438-4221-00205 12398216

[B50] Stein M Rappuoli R Covacci A Tyrosine phosphorylation of the *Helicobacter pylori cag*A antigen after *cag*-driven host cell translocation Proc Natl Acad Sci USA 2000 97 3 1263 8 10.1073/pnas.97.3.1263 10655519 PMC15590

[B51] Eppinger M Baar C Linz B Raddatz G Lanz C Keller H Morelli G Gressmann H Achtman M Schuster SC Who ate whom? Adaptive Helicobacter genomic changes that accompanied a host jump from early humans to large felines PLoS Genet 2006 2 e120 10.1371/journal.pgen.0020120 16789826 PMC1523251

[B52] Wicker T Schlagenhauf E Graner A Close T Keller B Stein N 454 sequencing put to the test using the complex genome of barley BMC Genomics 2006 7 275 10.1186/1471-2164-7-275 17067373 PMC1633745

[B53] Worst D Otto B de Graaff J Iron-repressible outer membrane proteins of *Helicobacter pylori *involved in heme uptake Infect Immun 1995 63 10 4161 5 7558334 10.1128/iai.63.10.4161-4165.1995 PMC173585

[B54] Velayudhan J Hughes N McColm A Bagshaw J Clayton C Andrews S Kelly D Iron acquisition and virulence in *Helicobacter pylori*: a major role for FeoB, a high-affinity ferrous iron transporter Mol Microbiol 2000 37 2 274 86 10.1046/j.1365-2958.2000.01987.x 10931324

[B55] Jönsson K Guo BP Monstein HJ Mekalanos J Kronvall G Molecular cloning and characterization of two *Helicobacter pylori *genes coding for plasminogen-binding proteins Proc Natl Acad Sci USA 2004 101 1852 1857 10.1073/pnas.0307329101 14769936 PMC357016

[B56] Lähteenmäki K Edelman S Korhonen TK Bacterial metastasis: the host plasminogen system in bacterial invasion Trends Microbiol 2005 13 79 85 10.1016/j.tim.2004.12.003 15680767

[B57] Carver TJ Rutherford KM Berriman M Rajandream MA Barrell BG Parkhill J ACT: the Artemis Comparison Tool Bioinformatics 2005 21 3422 3423 10.1093/bioinformatics/bti553 15976072

[B58] Laboratory of Phil Green http://www.phrap.org

[B59] Staden R Beal K Bonfield J The Staden package, 1998 Methods Mol Biol 2000 132 115 30 10547834 10.1385/1-59259-192-2:115

[B60] Meyer F Goesmann A McHardy A Bartels D Bekel T Clausen J Kalinowski J Linke B Rupp O Giegerich R Pühler A GenDB-an open source genome annotation system for prokaryote genomes Nucleic Acids Res 2003 31 8 2187 95 10.1093/nar/gkg312 12682369 PMC153740

[B61] Kurtz S Phillippy A Delcher A Smoot M Shumway M Antonescu C Salzberg S Versatile and open software for comparing large genomes Genome Biol 2004 5 2 R12 10.1186/gb-2004-5-2-r12 14759262 PMC395750

[B62] Richter D Schuster S Huson D OSLay: optimal syntenic layout of unfinished assemblies Bioinformatics 2007 23 13 1573 9 10.1093/bioinformatics/btm153 17463020

[B63] The Vmatch large scale sequence analysis software http://www.vmatch.de

[B64] Ussery DW Binnewies TT Gouveia-Oliveira R Jarmer H Hallin PF Genome update: DNA repeats in bacterial genomes Microbiology 2004 150 3519 3521 10.1099/mic.0.27628-0 15528640

[B65] Correspondence Analysis of Codon Usage http://codonw.sourceforge.net/

[B66] R Development Core Team R: A Language and Environment for Statistical Computing 2009 R Foundation for Statistical Computing, Vienna, Austria http://www.R-project.org [ISBN 3-900051-07-0]

[B67] Steinbiss S Gremme G Schärfer C Mader M Kurtz S AnnotationSketch: a genome annotation drawing library Bioinformatics 2009 25 4 533 534 10.1093/bioinformatics/btn657 19106120

