# Supplementary material for: Sequencing, annotation, and comparative genome analysis of the gerbil-adapted Helicobacter pylori strain B8
Source: BMC Genomics. 2010 May 27;11:335. doi: 10.1186/1471-2164-11-335 (PMC3091624; doi:10.1186/1471-2164-11-335)
Supplement: Additional file 1 — This file contains 11 tables and 7 figures with additional information. Here is a list of abbreviations of the table and figure captions with the page numbers in Additional file 1 where they start: Supplementary Tables S1 Regions of strain B8 covered by supercontigs of strain B128 1 S2 Pairs of B128-supercontigs with overlaps 4 S3 List of genes in uncovered regions of strain B8 5 S4 Distribution of the number of B8 proteins with no hit in strain B128 7 S5 List of strong singletons 8 S6 List of weak singletons 18 S7 List of singletons of strain B128 vs. strain B8 20 S8 List of singletons of strain B8 vs. four reference strains 21 S9 List of singletons of strain B8 versus strains J99 and P12 29 S10 List of singletons of strain B8 versus strains 26695 and HPAG1 30 S11 List of coding sequences in the plasticity zone of strain B8 32 Supplementary Figures S1 Distribution of the repeat density for 1053 bacterial genomes 35 S2 Comparison of genes in the plasticity zones of four H. pylori strains 36 S3 Synteny plot of strain B8 vs. strain J99 37 S4 Synteny plot of strain B8 vs. strain P12 37 S5 Distribution of relative codon usage 39 S6 Codon usage of four groups of B8 genes 40 S7 Codon usage of strain B8, H. acinonychis Sheeba, and E. coli K12 41 [file 1471-2164-11-335-S1.PDF]

Additional file 1

for the manuscript

Sequencing, annotation, and comparative genome analysis of the gerbil-adapted *Helicobacter pylori* strain B8

May 20, 2010

Table S1: Regions of the genome of strain B8 covered by supercontigs of strain B128.

B128 supercontig	B128 supercontig length	first matching position in B8	last matching position in B8	length of match in B8	first matching position in B128	last matching position in B128	length of match in B128	sequence identity	supercontig coverage
contig00199	85815	0	85475	85476	0	85482	85483	99.96	0.9961
contig00167	2693	85655	88346	2692	0	2692	2693	99.96	1.0000
contig00027	8651	88348	96997	8650	0	8650	8651	99.94	1.0000
contig00016	70725	97000	167718	70719	0	70723	70724	99.84	1.0000
contig00132	42941	167824	210758	42935	0	42940	42941	99.98	1.0000
contig00129	787	212317	213103	787	0	786	787	100.00	1.0000
contig00150	3829	213511	217340	3830	0	3828	3829	99.97	1.0000
contig00002	55406	217355	272761	55407	0	55405	55406	99.93	1.0000
contig00004	13227	273868	287091	13224	0	13226	13227	99.98	1.0000
contig00172	33934	289151	323082	33932	0	33933	33934	99.96	1.0000
contig00202	26421	325109	351524	26416	0	26420	26421	99.97	1.0000
contig00006	843	351693	352541	849	0	842	843	99.18	1.0000
contig00023	9874	355664	365538	9875	0	9873	9874	99.97	1.0000

Table S1 – continued from previous page

B128 supercontig	B128 supercontig length	first matching position in B8	last matching position in B8	length of match in B8	first matching position in B128	last matching position in B128	length of match in B128	sequence identity	supercontig coverage
contig00177	887	365622	366508	887	0	886	887	100.00	1.0000
contig00159	29876	366509	396384	29876	0	29875	29876	99.97	1.0000
contig00142	47434	397672	445105	47434	0	47433	47434	99.89	1.0000
contig00188	2348	445387	447734	2348	0	2347	2348	100.00	1.0000
contig00196	4507	447733	452247	4515	0	4506	4507	99.62	1.0000
contig00065	13511	452278	465785	13508	0	13510	13511	99.96	1.0000
contig00192	3156	465787	468941	3155	0	3155	3156	99.97	1.0000
contig00026	22813	469141	491949	22809	0	22812	22813	99.97	1.0000
contig00190	7186	494663	501858	7196	0	7185	7186	90.16	1.0000
contig00011	29341	501975	527522	25548	3792	29340	25549	99.57	0.8708
contig00019	27121	528790	555917	27128	0	27115	27116	98.91	0.9998
contig00158	24526	556170	580682	24513	0	24524	24525	99.91	1.0000
contig00020	2197	580687	582884	2198	0	2196	2197	99.91	1.0000
contig00008	8121	582885	591004	8120	0	8120	8121	99.99	1.0000
contig00175	36978	591386	628402	37017	5	36977	36973	98.70	0.9999
contig00147	17730	631639	649365	17727	0	17729	17730	99.95	1.0000
contig00010	18405	649589	667997	18409	0	18404	18405	99.95	1.0000
contig00198	22120	667994	690108	22115	0	22119	22120	99.98	1.0000
contig00121	1064	691052	692114	1063	0	1063	1064	99.91	1.0000
contig00141	17220	693878	711096	17219	0	17219	17220	99.95	1.0000
contig00184	4419	711100	715518	4419	0	4418	4419	100.00	1.0000
contig00160	4508	716011	720518	4508	0	4507	4508	100.00	1.0000
contig00025	38448	721279	759718	38440	2	38447	38446	99.96	0.9999
contig00187	72944	759734	832670	72937	0	72942	72943	99.96	1.0000
contig00022	911	833173	834084	912	0	910	911	99.56	1.0000
contig00156	35539	834091	869626	35536	0	35538	35539	99.97	1.0000
contig00194	6207	869732	875937	6206	0	6206	6207	99.95	1.0000
contig00201	26113	875863	901964	26102	0	26112	26113	99.95	1.0000
contig00173	4303	904163	908464	4302	0	4302	4303	99.98	1.0000
contig00162	4028	908392	912418	4027	0	4026	4027	100.00	0.9998
contig00139	2055	912419	914474	2056	1	2054	2054	99.81	0.9995
contig00005	12791	914475	927272	12798	0	12790	12791	99.89	1.0000
contig00186	57451	927206	984649	57444	2	57450	57449	99.96	1.0000
contig00152	18648	985299	1003937	18639	0	18647	18648	99.94	1.0000
contig00180	34347	1003948	1038288	34341	0	34346	34347	99.96	1.0000
contig00149	749	1038290	1039038	749	0	748	749	100.00	1.0000
contig00009	649	1039041	1039688	648	0	646	647	99.85	0.9969
contig00151	13292	1039694	1052980	13287	0	13291	13292	99.96	1.0000
contig00148	12096	1053024	1065116	12093	0	12095	12096	99.98	1.0000
contig00183	5915	1065126	1071040	5915	0	5914	5915	100.00	1.0000
contig00193	12815	1071207	1084016	12810	0	12814	12815	99.93	1.0000
contig00021	226574	1084395	1311035	226641	0	226573	226574	95.13	1.0000
contig00176	21399	1311039	1332438	21400	0	21398	21399	99.97	1.0000

Table S1 – continued from previous page

B128 supercontig	B128 supercontig length	first matching position in B8	last matching position in B8	length of match in B8	first matching position in B128	last matching position in B128	length of match in B128	sequence identity	supercontig coverage
contig00003	82147	1332459	1414588	82130	0	82144	82145	99.95	1.0000
contig00197	22811	1414296	1437120	22825	0	22800	22801	98.88	0.9996
contig00001	19297	1437208	1456509	19302	0	19296	19297	99.95	1.0000
contig00185	6604	1456510	1463100	6591	0	6603	6604	99.74	1.0000
contig00171	6865	1462986	1469840	6855	0	6863	6864	99.81	0.9999
contig00165	20875	1469841	1490715	20875	0	20874	20875	99.98	1.0000
contig00189	798	1490718	1491515	798	0	796	797	99.62	0.9987
contig00164	37192	1491517	1528696	37180	0	37191	37192	99.95	1.0000
contig00013	4947	1533305	1538251	4947	0	4946	4947	100.00	1.0000
contig00014	4614	1538534	1543146	4613	0	4613	4614	99.89	1.0000
contig00155	95730	1543942	1639669	95728	0	95729	95730	99.95	1.0000
contig00182	17605	1639673	1657270	17598	0	17604	17605	99.94	1.0000
contig00163	2529	1657289	1659817	2529	0	2528	2529	99.96	1.0000
contig00179	10263	1659823	1670082	10260	0	10262	10263	99.97	1.0000
contig00154	3116	1670546	1673661	3116	0	3115	3116	100.00	1.0000

Table S2: Pairs of B128-supercontigs with overlaps. The end of the first contig overlaps with the beginning of the second supercontig by the given number of bp. The pairs are ordered by the position where they match in the genome of strain B8.

supercontig1	length of supercontig1 (bp)	supercontig2	length of supercontig2 (bp)	length of overlap (bp)
contig00188	2348	contig00196	4507	2
contig00010	18405	contig00198	22120	4
contig00194	6207	contig00201	26113	75
contig00173	4303	contig00162	4028	73
contig00005	12791	contig00186	57451	67
contig00003	82147	contig00197	22811	293
contig00185	6604	contig00171	6865	115

Table S3: List of 60 genes in uncovered regions of strain B8. In green we show the genes for which there is no complete match with at most 2% differences on the DNA level and no 80/80 blastn hit in the B128-supercontigs (i.e. weak singletons).

	position1	positions2	length	gene	product
1	85477	85655	179	HPB8_81	hypothetical protein predicted by Glimmer/Critica
2	96999	97000	2	HPB8_96	conserved hypothetical protein
3	167720	167824	105	HPB8_193	hypothetical protein
4	210760	212317	1558	HPB8_237	blood group antigen-binding adhesin BabA fragment
	213105	213511	407	HPB8_237	blood group antigen-binding adhesin BabA fragment
5	213105	213511	407	HPB8_238	hypothetical protein predicted by Glimmer/Critica
6	272763	273868	1106	HPB8_303	conserved hypothetical protein
7	287093	289151	2059	HPB8_319	conserved hypothetical protein
8	351526	351693	168	HPB8_386	hypothetical protein
	352543	355664	3122	HPB8_386	hypothetical protein
9	352543	355664	3122	HPB8_387	hypothetical protein
10	352543	355664	3122	HPB8_388	hypothetical protein
11	352543	355664	3122	HPB8_389	conserved hypothetical protein
12	396386	397672	1287	HPB8_426	bifunctional cytochrome c biogenesis protein
13	396386	397672	1287	HPB8_427	Alpha-(1,3)-fucosyltransferase 11
14	396386	397672	1287	HPB8_428	fucosyltransferase
15	445107	445387	281	HPB8_475	hypothetical protein
16	465787	465787	1	HPB8_497	type IV secretion system protein VirB11
17	468943	469141	199	HPB8_502	hypothetical protein predicted by Glimmer/Critica
18	491951	494663	2713	HPB8_519	conserved hypothetical protein, partial cds
19	491951	494663	2713	HPB8_520	hypothetical protein predicted by Glimmer/Critica
20	491951	494663	2713	HPB8_521	conserved hypothetical protein
21	501860	501975	116	HPB8_529	conserved hypothetical protein
22	527524	528790	1267	HPB8_556	integrase/recombinase (XerCD family)
23	527524	528790	1267	HPB8_557	hypothetical protein predicted by Glimmer/Critica
24	555919	556170	252	HPB8_582	Interferon-induced GTP-binding protein Mx2
25	555919	556170	252	HPB8_583	hypothetical protein
26	591006	591386	381	HPB8_626	conserved hypothetical protein
27	628404	631639	3236	HPB8_657	blood group antigen-binding adhesin BabA
28	628404	631639	3236	HPB8_658	hypothetical protein predicted by Glimmer/Critica
29	649367	649589	223	HPB8_674	conserved hypothetical protein
30	690110	691052	943	HPB8_716	cag pathogenicity island protein Y VirB10-like protein
	692116	693878	1763	HPB8_716	cag pathogenicity island protein Y VirB10-like protein
31	711098	711100	3	HPB8_733	GTP-binding protein Era
32	715520	716011	492	HPB8_739	Regulator of nonsense transcripts 1
33	720520	721279	760	HPB8_742	Regulator of nonsense transcripts 1 homolog
34	832672	833173	502	HPB8_853	Chromosomal replication initiator protein dnaA

Table S3 – continued from previous page

	position1	positions2	length	gene	product
35	832672	833173	502	HPB8_854	Alpha-(1,3)-fucosyltransferase 11
36	869628	869732	105	HPB8_884	conserved hypothetical protein
37	901966	904163	2198	HPB8_915	putative outer membrane protein
38	984651	985299	649	HPB8_998	type I restriction enzyme, S subunit
39	1038290	1038290	1	HPB8_1056	type I restriction enzyme, R subunit
40	1039040	1039041	2	HPB8_1057	type I restriction enzyme, S subunit
41	1039690	1039694	5	HPB8_1058	type I restriction enzyme M protein
42	1052982	1053024	43	HPB8_1072	plasminogen-binding protein pgbB
43	1071042	1071207	166	HPB8_1092	conserved hypothetical protein
44	1071042	1071207	166	HPB8_1093	hypothetical protein predicted by Glimmer/Critica
45	1084018	1084395	378	HPB8_1104	conserved hypothetical protein
46	1437122	1437208	87	HPB8_1467	methyl-accepting chemotaxis protein
47	1490717	1490718	2	HPB8_1521	adenine-specific DNA-methyltransferase
48	1491517	1491517	1	HPB8_1522	putative agmatine deiminase
49	1528698	1533305	4608	HPB8_1557	hypothetical protein predicted by Glimmer/Critica
50	1528698	1533305	4608	HPB8_1558	hypothetical protein predicted by Glimmer/Critica
51	1528698	1533305	4608	HPB8_1559	hypothetical protein predicted by Glimmer/Critica
52	1528698	1533305	4608	HPB8_1560	hypothetical protein predicted by Glimmer/Critica
53	1528698	1533305	4608	HPB8_1561	hypothetical protein
54	1538253	1538534	282	HPB8_1567	conserved hypothetical protein
55	1538253	1538534	282	HPB8_1568	hypothetical protein
56	1543148	1543942	795	HPB8_1575	adenine DNA methyltransferase protein
57	1543148	1543942	795	HPB8_1576	agmatine deiminase
58	1639671	1639673	3	HPB8_1680	Na+:H+ antiporter, NhaA family
59	1670084	1670546	463	HPB8_1707	hypothetical protein predicted by Glimmer/Critica
60	1670084	1670546	463	HPB8_1708	hypothetical protein predicted by Glimmer/Critica

Table S4: Distribution of the number of B8-proteins with no blastp hit in B128 according to the parameters given in the first two columns. The fourth column shows the number of singletons with an exact match on the DNA level. The fifth column is the ratio of the values in column 4 and column 5.

identity in %	coverage in %	number of singletons	exact cds	ratio
100	100	673	614	0.91
100	95	622	564	0.91
100	90	588	530	0.90
100	85	564	506	0.90
100	80	548	490	0.89
100	75	537	479	0.89
100	70	526	468	0.89
95	100	630	573	0.91
95	95	549	493	0.90
95	90	490	434	0.89
95	85	456	400	0.88
95	80	432	376	0.87
95	75	402	347	0.86
95	70	376	323	0.86
90	100	630	573	0.91
90	95	548	492	0.90
90	90	488	432	0.89
90	85	454	398	0.88
90	80	429	373	0.87
90	75	398	343	0.86
90	70	372	319	0.86
85	100	629	572	0.91
85	95	547	491	0.90
85	90	487	431	0.89
85	85	453	397	0.88
85	80	428	372	0.87
85	75	397	342	0.86
85	70	371	318	0.86
80	100	628	572	0.91
80	95	546	491	0.90
80	90	485	431	0.89
80	85	450	396	0.88
• 80	80	425	371	0.87
80	75	394	341	0.87
80	70	369	317	0.86
75	100	627	571	0.91
75	95	545	490	0.90
75	90	484	430	0.89
75	85	448	395	0.88
75	80	423	370	0.87
75	75	391	339	0.87
75	70	366	315	0.86
70	100	627	571	0.91
70	95	544	489	0.90
70	90	484	430	0.89
70	85	448	395	0.88
70	80	423	370	0.87
70	75	391	339	0.87
70	70	366	315	0.86

Table S5: List of 371 genes of strain B8 with a complete match of at most 2% differences on the DNA level but no 80/80 blastp hit on the protein level, all with respect to the B128 genome. The last two columns show the locus tags of all genes in strain J99 and strain 26695 which are ortholog to the given gene of strain B8.

	coding sequence	product	J99-ortho	26695-ortho
1	HPB8_2	conserved hypothetical protein	jhp1416	HP1527
2	HPB8_7	site-specific DNA-methyltransferase (adenine-specific)		
3	HPB8_9	type III restriction enzyme	jhp1410	HP1521
4	HPB8_10	putative type IIS restriction/modification enzyme		
5	HPB8_16	hypothetical protein predicted by Glimmer/Critica		
6	HPB8_23	glutamate:Na+ symporter, ESS family	jhp1399	HP1506
7	HPB8_35	transaldolase	jhp1388	HP1495
8	HPB8_51	conserved hypothetical protein	jhp1372	HP1479
9	HPB8_59	conserved hypothetical protein		
10	HPB8_61	DNA polymerase I	jhp1363	HP1470
11	HPB8_63	branched-chain amino acid aminotransferase	jhp1361	HP1468
12	HPB8_70	hypothetical protein predicted by Glimmer/Critica		
13	HPB8_73	ribosomal large subunit pseudouridine synthase B	jhp1352	HP1459
14	HPB8_75	hypothetical protein predicted by Glimmer/Critica		
15	HPB8_76	hypothetical protein predicted by Glimmer/Critica		
16	HPB8_82	hypothetical protein predicted by Glimmer/Critica		
17	HPB8_86	preprotein translocase YidC subunit	jhp1343	HP1450
18	HPB8_93	4-diphosphocytidyl-2-C-methyl-D-erythritolkinase	jhp1336	HP1443
19	HPB8_96	conserved hypothetical protein	jhp1333	HP1440
20	HPB8_100	hypothetical protein predicted by Glimmer/Critica		
21	HPB8_102	hypothetical protein		
22	HPB8_104	hypothetical protein predicted by Glimmer/Critica		
23	HPB8_108	type I R-M system specificity subunit		
24	HPB8_109	hypothetical protein predicted by Glimmer/Critica		
25	HPB8_110	hypothetical protein predicted by Glimmer/Critica		
26	HPB8_111	hypothetical protein predicted by Glimmer/Critica		
27	HPB8_146	hypothetical protein predicted by Glimmer/Critica		
28	HPB8_151	conserved hypothetical protein	jhp1248	HP1328
29	HPB8_158	conserved hypothetical protein	jhp1241	HP1321
30	HPB8_169	small subunit ribosomal protein S17	jhp1230	HP1310
31	HPB8_173	30S ribosomal protein S14 type Z	jhp1226	HP1306
32	HPB8_182	50S ribosomal protein L36	jhp1217	HP1297
33	HPB8_188	hypothetical protein predicted by Glimmer/Critica		
34	HPB8_189	hypothetical protein predicted by Glimmer/Critica		
35	HPB8_190	thiamine pyrophosphokinase	jhp1211	HP1291
36	HPB8_198	anthranilate synthase component I	jhp1203	HP1282
37	HPB8_209	NADH dehydrogenase I chain L	jhp1192	HP1271

Table S5 – continued from previous page

	coding sequence	product	J99-ortho	26695-ortho
38	HPB8_213	NADH dehydrogenase I chain H	jhp1188	HP1267
39	HPB8_214	NADH dehydrogenase I chain G	jhp1187	HP1266
40	HPB8_222	conserved hypothetical protein	jhp1179	HP1258
41	HPB8_223	orotate phosphoribosyltransferase	jhp1178	HP1257
42	HPB8_233	DNA polymerase III subunit delta	jhp1168	HP1247
43	HPB8_239	hypothetical protein predicted by Glimmer/Critica		
44	HPB8_240	hypothetical protein predicted by Glimmer/Critica		
45	HPB8_242	alanyl-tRNA synthetase	jhp1162	HP1241
46	HPB8_244	hypothetical protein predicted by Glimmer/Critica		
47	HPB8_245	hypothetical protein predicted by Glimmer/Critica		
48	HPB8_248	conserved hypothetical protein	jhp1157	HP1236
49	HPB8_251	hypothetical protein predicted by Glimmer/Critica		
50	HPB8_266	hypothetical protein predicted by Glimmer/Critica		
51	HPB8_267	hypothetical protein predicted by Glimmer/Critica		
52	HPB8_268	conserved hypothetical protein		HP1388
53	HPB8_269	hypothetical protein predicted by Glimmer/Critica		
54	HPB8_271	hypothetical protein predicted by Glimmer/Critica		
55	HPB8_275	polyribonucleotide nucleotidyltransferase	jhp1136	HP1213
56	HPB8_281	adenine-specific DNA-methyltransferase	jhp1131	HP1208
57	HPB8_282	hypothetical protein predicted by Glimmer/Critica		
58	HPB8_286	50S ribosomal protein L33	jhp1127	HP1204
59	HPB8_289	large subunit ribosomal protein L11	jhp1125	HP1202
60	HPB8_293	hypothetical protein predicted by Glimmer/Critica		
61	HPB8_298	hypothetical protein predicted by Glimmer/Critica		
62	HPB8_300	heptosyltransferase II	jhp1116	HP1191
63	HPB8_305	hypothetical protein predicted by Glimmer/Critica		
64	HPB8_307	hypothetical protein predicted by Glimmer/Critica		
65	HPB8_317	hypothetical protein predicted by Glimmer/Critica		
66	HPB8_318	hypothetical protein predicted by Glimmer/Critica		
67	HPB8_319	conserved hypothetical protein		
68	HPB8_320	hypothetical protein predicted by Glimmer/Critica		
69	HPB8_323	hypothetical protein predicted by Glimmer/Critica		
70	HPB8_325	putative glutamine transport system substrate-binding protein	jhp1099	HP1172
71	HPB8_332	conserved hypothetical protein	jhp1092	HP1165
72	HPB8_335	hypothetical protein predicted by Glimmer/Critica		
73	HPB8_337	flavodoxin I	jhp1088	HP1161
74	HPB8_344	hypothetical protein predicted by Glimmer/Critica		
75	HPB8_347	hypothetical protein predicted by Glimmer/Critica		
76	HPB8_349	signal recognition particle, subunit SRP54	jhp1079	HP1152
77	HPB8_356	hypothetical protein		

Table S5 – continued from previous page

	coding sequence	product	J99-ortho	26695-ortho
78	HPB8_357	hypothetical protein predicted by Glimmer/Critica		
79	HPB8_360	conserved hypothetical protein		
80	HPB8_378	hypothetical protein predicted by Glimmer/Critica		
81	HPB8_380	DNA (cytosine-5-)-methyltransferase	jhp1050	HP1121
82	HPB8_383	conserved hypothetical protein		
83	HPB8_387	hypothetical protein		
84	HPB8_396	pyruvate ferredoxin oxidoreductase, gamma subunit	jhp1035	HP1108
85	HPB8_403	glucose-6-phosphate 1-dehydrogenase	jhp1027	HP1101
86	HPB8_409	UDP-glucose 4-epimerase	jhp1020	HP0360
87	HPB8_411	conserved hypothetical protein	jhp1018	HP0362
88	HPB8_414	spore coat polysaccharide biosynthesis protein C	jhp1015	HP0366
89	HPB8_417	putative type II DNA modification enzyme (methyltransferase)	jhp1012	
90	HPB8_426	bifunctional cytochrome c biogenesis protein	jhp1003	HP0378
91	HPB8_433	conserved hypothetical protein	jhp0997	HP0384
92	HPB8_434	hypothetical protein predicted by Glimmer/Critica		
93	HPB8_437	primosomal protein N' (replication factor Y) (superfamily II helicase)	jhp0994	HP0387
94	HPB8_443	putative chemotaxis protein		
95	HPB8_449	hypothetical protein	jhp0983	HP0398
96	HPB8_451	4-hydroxy-3-methylbut-2-enyl diphosphate reductase	jhp0981	HP0400
97	HPB8_452	3-phosphoshikimate 1-carboxyvinyltransferase	jhp0980	HP0401
98	HPB8_455	hypothetical protein predicted by Glimmer/Critica		
99	HPB8_462	hypothetical protein predicted by Glimmer/Critica		
100	HPB8_464	hypothetical protein predicted by Glimmer/Critica	jhp0970	HP0412
101	HPB8_469	tRNA (mo5U34)-methyltransferase	jhp0965	HP0419
102	HPB8_473	conserved hypothetical protein	jhp0961	
103	HPB8_478	hypothetical protein predicted by Glimmer/Critica		
104	HPB8_479	hypothetical protein predicted by Glimmer/Critica		
105	HPB8_480	hypothetical protein predicted by Glimmer/Critica		
106	HPB8_481	hypothetical protein predicted by Glimmer/Critica		
107	HPB8_482	conserved hypothetical protein		HP0446
108	HPB8_485	DNA transfer protein		
109	HPB8_487	DNA topoisomerase I		HP0440
110	HPB8_491	hypothetical pseudogene		
111	HPB8_495	conserved hypothetical protein		
112	HPB8_496	conserved hypothetical protein		
113	HPB8_500	hypothetical protein predicted by Glimmer/Critica		
114	HPB8_504	conserved hypothetical protein		HP1001
115	HPB8_512	conserved hypothetical protein	jhp0947	
116	HPB8_516	hypothetical protein predicted by Glimmer/Critica		
117	HPB8_518	putative transposase		

Table S5 – continued from previous page

	coding sequence	product	J99-ortho	26695-ortho
118	HPB8_520	hypothetical protein predicted by Glimmer/Critica		
119	HPB8_528	conserved hypothetical protein		
120	HPB8_533	hypothetical protein		
121	HPB8_543	conserved hypothetical protein		
122	HPB8_547	hypothetical protein predicted by Glimmer/Critica		
123	HPB8_548	type IV secretion system protein VirB4		HP0459
124	HPB8_559	hypothetical protein predicted by Glimmer/Critica		
125	HPB8_560	hypothetical protein predicted by Glimmer/Critica		
126	HPB8_562	hypothetical protein predicted by Glimmer/Critica		
127	HPB8_565	hypothetical protein predicted by Glimmer/Critica		
128	HPB8_570	hypothetical protein predicted by Glimmer/Critica		
129	HPB8_574	glycyl-tRNA synthetase beta chain	jhp0906	HP0972
130	HPB8_577	Multidrug resistance protein mdtB	jhp0903	HP0969
131	HPB8_578	conserved hypothetical protein	jhp0902	
132	HPB8_581	probable tRNA modification GTPase trmE		
133	HPB8_585	Glycyl-tRNA synthetase, subunit alpha	jhp0894	HP0960
134	HPB8_589	hypothetical protein predicted by Glimmer/Critica		
135	HPB8_598	conserved hypothetical protein	jhp0882	HP0948
136	HPB8_599	hypothetical protein predicted by Glimmer/Critica		
137	HPB8_600	hypothetical protein predicted by Glimmer/Critica		
138	HPB8_601	hypothetical protein predicted by Glimmer/Critica		
139	HPB8_602	conserved hypothetical protein	jhp0881	HP0947
140	HPB8_603	conserved hypothetical protein	jhp0880	HP0946
141	HPB8_611	conserved hypothetical protein		
142	HPB8_612	hypothetical protein predicted by Glimmer/Critica		
143	HPB8_613	conserved hypothetical protein	jhp0871	HP0936
144	HPB8_614	putative outer membrane protein		
145	HPB8_633	hypothetical protein predicted by Glimmer/Critica		
146	HPB8_635	hypothetical protein predicted by Glimmer/Critica		
147	HPB8_640	hypothetical protein predicted by Glimmer/Critica		
148	HPB8_646	conserved hypothetical protein	jhp0842	HP0906
149	HPB8_647	phosphate acetyltransferase	jhp0841	
150	HPB8_654	Hydrogenase expression/formation protein hypD2	jhp0835	HP0898
151	HPB8_664	iron compounds ABC transporter, ATP-binding protein	jhp0821	HP0888
152	HPB8_668	integral membrane protein MviN	jhp0817	HP0885
153	HPB8_672	hypothetical protein		
154	HPB8_679	UDP-N-acetylmuramoyl-L-alanine:D-glutamate ligase (ADP-forming)	jhp0446	HP0494
155	HPB8_684	outer membrane phospholipase A1 precursor	jhp0451	HP0499
156	HPB8_688	hypothetical protein		
157	HPB8_689	hypothetical protein		

	coding sequence	product	J99-ortho	26695-ortho
158	HPB8_695	hypothetical protein predicted by Glimmer/Critica		
159	HPB8_700	cag pathogenicity island protein F	jhp0491	HP0543
160	HPB8_707	hypothetical protein predicted by Glimmer/Critica		
161	HPB8_708	hypothetical protein		
162	HPB8_714	cag pathogenicity island protein W	jhp0478	HP0529
163	HPB8_715	cag pathogenicity island protein X VirB9-like protein	jhp0477	HP0528
164	HPB8_720	hypothetical protein predicted by Glimmer/Critica		
165	HPB8_721	hypothetical protein predicted by Glimmer/Critica		
166	HPB8_724	cag pathogenicity island protein Epsilon		
167	HPB8_727	hypothetical protein predicted by Glimmer/Critica		
168	HPB8_729	conserved hypothetical protein		
169	HPB8_735	conserved hypothetical protein		
170	HPB8_746	conserved hypothetical protein	jhp0499	HP0552
171	HPB8_747	RNA methyltransferase, TrmH family	jhp0500	HP0553
172	HPB8_751	hypothetical protein predicted by Glimmer/Critica		
173	HPB8_752	hypothetical protein predicted by Glimmer/Critica		
174	HPB8_759	hypothetical protein predicted by Glimmer/Critica		
175	HPB8_762	Diaminopimelate epimerase	jhp0513	HP0566
176	HPB8_772	signal peptidase I	jhp0523	HP0576
177	HPB8_774	phosphatidylglycerol-membrane-oligosaccharide glycerophosphotransferase MdoB	jhp0525	HP0578
178	HPB8_777	hypothetical protein		
179	HPB8_780	conserved hypothetical protein	jhp0530	HP0583
180	HPB8_799	multidrug resistance protein (SpaB)		
181	HPB8_801	Endonuclease III	jhp0549	HP0602
182	HPB8_809	putative ABC-2 type transport system permease protein	jhp0299	
183	HPB8_811	hypothetical protein predicted by Glimmer/Critica		
184	HPB8_813	NAD-dependent DNA ligase LigA	jhp0558	HP0615
185	HPB8_818	jhp0563-like glycosyltransferase, family 25		
186	HPB8_827	hypothetical protein predicted by Glimmer/Critica		
187	HPB8_830	hypothetical protein predicted by Glimmer/Critica		
188	HPB8_835	conserved hypothetical protein	jhp0578	HP0635
189	HPB8_838	outer inflammatory protein OipA	jhp0581	HP0638
190	HPB8_840	hypothetical protein predicted by Glimmer/Critica		
191	HPB8_841	Polynucleotide adenylyltransferase(PapS)	jhp0583	HP0640
192	HPB8_858	putative outer membrane protein	jhp0600	HP0655
193	HPB8_875	hypothetical protein predicted by Glimmer/Critica		
194	HPB8_885	bifunctional protein glmU	jhp0624	HP0683
195	HPB8_891	3-oxoacid CoA-transferase, subunit A	jhp0637	HP0691
196	HPB8_895	5-oxoprolinase		
197	HPB8_899	hypothetical protein predicted by Glimmer/Critica		

	coding sequence	product	J99-ortho	26695-ortho
198	HPB8_906	hypothetical protein predicted by Glimmer/Critica		
199	HPB8_908	conserved hypothetical protein		
200	HPB8_911	hypothetical protein predicted by Glimmer/Critica		
201	HPB8_915	putative outer membrane protein	jhp0649	HP0710
202	HPB8_917	hypothetical protein		
203	HPB8_927	Mesentericin Y105 secretion protein mesE	jhp1103	HP1177
204	HPB8_940	hypothetical protein		
205	HPB8_941	conserved hypothetical protein	jhp0671	HP0734
206	HPB8_954	tRNA (guanine-N7-)-methyltransferase	jhp0684	HP0747
207	HPB8_962	conserved hypothetical protein	jhp0692	HP0755
208	HPB8_975	hypothetical protein predicted by Glimmer/Critica		
209	HPB8_978	molybdopterin-guanine dinucleotide biosynthesis protein A	jhp0706	HP0769
210	HPB8_979	flagellar biosynthetic protein FlhB	jhp0707	HP0770
211	HPB8_987	conserved hypothetical protein	jhp0715	HP0778
212	HPB8_989	conserved hypothetical protein	jhp0717	HP0780
213	HPB8_994	preprotein translocase SecA subunit	jhp0723	HP0786
214	HPB8_995	conserved hypothetical protein	jhp0724	HP0787
215	HPB8_997	hypothetical protein predicted by Glimmer/Critica		
216	HPB8_1003	trigger factor	jhp0731	HP0795
217	HPB8_1005	neuraminyllactose-binding hemagglutinin [Precursor]	jhp0733	HP0797
218	HPB8_1015	iron complex outermembrane recepter protein	jhp0743	HP0807
219	HPB8_1019	conserved hypothetical protein	jhp0747	HP0811
220	HPB8_1022	thiamine biosynthesis protein ThiF	jhp0750	HP0814
221	HPB8_1026	Osmoprotection protein (ProWX)	jhp0757	HP0818
222	HPB8_1028	hypothetical protein predicted by Glimmer/Critica		
223	HPB8_1042	hypothetical protein predicted by Glimmer/Critica		
224	HPB8_1046	hypothetical protein predicted by Glimmer/Critica		
225	HPB8_1058	type I restriction enzyme M protein	jhp0786	HP0850
226	HPB8_1060	type II restriction endonuclease		
227	HPB8_1061	conserved hypothetical protein	jhp0787	HP0851
228	HPB8_1062	conserved hypothetical protein	jhp0788	HP0852
229	HPB8_1065	hypothetical protein predicted by Glimmer/Critica		
230	HPB8_1077	conserved hypothetical protein	jhp0802	HP0868
231	HPB8_1080	hypothetical protein predicted by Glimmer/Critica		
232	HPB8_1083	hypothetical protein predicted by Glimmer/Critica		
233	HPB8_1094	conserved hypothetical protein	jhp0439	HP0487
234	HPB8_1097	conserved hypothetical protein		HP0484
235	HPB8_1105	hypothetical protein predicted by Glimmer/Critica		
236	HPB8_1107	molybdate transport system ATP-binding protein	jhp0427	HP0475
237	HPB8_1110	hypothetical protein predicted by Glimmer/Critica		

Table S5 – continued from previous page

	coding sequence	product	J99-ortho	26695-ortho
238	HPB8_1120	type I restriction enzyme R protein		
239	HPB8_1124	hypothetical protein predicted by Glimmer/Critica		
240	HPB8_1125	hypothetical protein predicted by Glimmer/Critica		
241	HPB8_1131	conserved hypothetical protein	jhp0408	HP1015
242	HPB8_1137	hypothetical protein predicted by Glimmer/Critica		
243	HPB8_1139	hypothetical protein		
244	HPB8_1140	hypothetical protein		
245	HPB8_1157	conserved hypothetical protein	jhp0385	HP1039
246	HPB8_1167	conserved hypothetical protein	jhp0376	HP1049
247	HPB8_1168	hypothetical protein predicted by Glimmer/Critica		
248	HPB8_1169	homoserine kinase	jhp0375	HP1050
249	HPB8_1176	conserved hypothetical protein	jhp0368	HP1057
250	HPB8_1180	sec-independent protein translocase protein TatC	jhp0364	HP1061
251	HPB8_1184	conserved hypothetical protein	jhp0360	HP1065
252	HPB8_1189	conserved hypothetical protein	jhp0355	HP1070
253	HPB8_1190	hypothetical protein predicted by Glimmer/Critica		
254	HPB8_1194	conserved hypothetical protein	jhp0350	HP1075
255	HPB8_1200	conserved hypothetical protein	jhp0344	HP1081
256	HPB8_1206	bifunctional riboflavin kinase/FMN adenylyltransferase	jhp0338	HP1087
257	HPB8_1209	DNA segregation ATPase FtsK/SpoIIIE, S-DNA-T family	jhp0335	HP1090
258	HPB8_1212	hypothetical protein predicted by Glimmer/Critica		
259	HPB8_1213	conserved hypothetical protein		
260	HPB8_1216	GTP-binding protein LepA	jhp0329	HP0355
261	HPB8_1222	hypothetical protein predicted by Glimmer/Critica		
262	HPB8_1224	single-stranded-DNA-specific exonuclease	jhp0322	HP0348
263	HPB8_1225	ribosomal large subunit pseudouridine synthase D	jhp0321	HP0347
264	HPB8_1226	conserved hypothetical protein		
265	HPB8_1228	hypothetical protein predicted by Glimmer/Critica		
266	HPB8_1229	Hcp beta-lactamase-like protein C1orf163		
267	HPB8_1242	hypothetical protein predicted by Glimmer/Critica		
268	HPB8_1243	conserved hypothetical protein		
269	HPB8_1248	hypothetical protein predicted by Glimmer/Critica		
270	HPB8_1249	conserved hypothetical protein	jhp0298	HP0313
271	HPB8_1255	conserved hypothetical protein	jhp0292	HP0307
272	HPB8_1258	conserved hypothetical protein	jhp0289	HP0304
273	HPB8_1260	dipeptide ABC transporter, ATP-binding protein (DppF)	jhp0287	HP0302
274	HPB8_1261	dipeptide ABC transporter, ATP-binding protein (DppD)	jhp0286	HP0301
275	HPB8_1262	dipeptide ABC transporter, permease protein (DppC)		HP0300
276	HPB8_1270	conserved hypothetical protein	jhp0277	HP0292

	coding sequence	product	J99-ortho	26695-ortho
277	HPB8_1273	conserved hypothetical protein	jhp0274	HP0289
278	HPB8_1289	conserved hypothetical protein	jhp0258	HP0273
279	HPB8_1292	conserved hypothetical protein	jhp0255	
280	HPB8_1296	dihydroorotase	jhp0251	HP0266
281	HPB8_1303	site-specific DNA-methyltransferase (adenine-specific)	jhp0244	HP0260
282	HPB8_1306	conserved hypothetical protein	jhp0241	HP0257
283	HPB8_1329	hypothetical protein predicted by Glimmer/Critica		
284	HPB8_1330	hypothetical protein predicted by Glimmer/Critica		
285	HPB8_1332	glutathionylspermidine synthase	jhp0218	HP0233
286	HPB8_1339	hypothetical protein predicted by Glimmer/Critica		
287	HPB8_1340	hypothetical protein predicted by Glimmer/Critica		
288	HPB8_1343	DNA repair protein RadA/Sms	jhp0209	HP0223
289	HPB8_1353	succinyl-diaminopimelate desuccinylase	jhp0198	HP0212
290	HPB8_1355	hypothetical protein predicted by Glimmer/Critica		
291	HPB8_1359	conserved hypothetical protein		
292	HPB8_1361	conserved hypothetical protein		
293	HPB8_1363	hypothetical protein predicted by Glimmer/Critica		
294	HPB8_1371	UDP-3-O-[3-hydroxymyristoyl] glucosamine N-acyltransferase	jhp0182	HP0196
295	HPB8_1377	conserved hypothetical protein	jhp0176	HP0190
296	HPB8_1380	hypothetical protein predicted by Glimmer/Critica		
297	HPB8_1385	membrane protein required for colicin V production	jhp0169	HP0181
298	HPB8_1398	conserved hypothetical protein	jhp0154	HP0168
299	HPB8_1402	porphobilinogen synthase	jhp0150	HP0163
300	HPB8_1404	hypothetical protein predicted by Glimmer/Critica		
301	HPB8_1423	A/G-specific adenine glycosylase	jhp0130	HP0142
302	HPB8_1426	(S)-2-hydroxy-acid oxidase	jhp0127	HP0139
303	HPB8_1432	hypothetical protein predicted by Glimmer/Critica		
304	HPB8_1435	hypothetical protein predicted by Glimmer/Critica		
305	HPB8_1437	hypothetical protein predicted by Glimmer/Critica		
306	HPB8_1439	hypothetical protein		HP0128
307	HPB8_1445	Uncharacterized protein jhp0112; Flags: Precursor		
308	HPB8_1446	pyruvate,water dikinase	jhp0111	HP0121
309	HPB8_1451	conserved hypothetical protein	jhp0106	HP0114
310	HPB8_1454	heat-inducible transcriptional repressor	jhp0103	HP0111
311	HPB8_1457	conserved hypothetical protein		HP0108
312	HPB8_1458	cysteine synthase	jhp0099	HP0107
313	HPB8_1461	2',3'-cyclic-nucleotide 2'-phosphodiesterase	jhp0096	HP0104
314	HPB8_1463	conserved hypothetical protein	jhp0094	HP0102
315	HPB8_1465	hypothetical protein predicted by Glimmer/Critica		
316	HPB8_1472	putative alpha(1,2)fucosyltransferase		

Table S5 – continued from previous page

	coding sequence	product	J99-ortho	26695-ortho
317	HPB8_1486	hypothetical protein predicted by Glimmer/Critica		
318	HPB8_1491	hypothetical protein predicted by Glimmer/Critica		
319	HPB8_1493	urease alpha subunit	jhp0067	HP0072
320	HPB8_1494	hypothetical protein predicted by Glimmer/Critica		
321	HPB8_1509	hypothetical protein predicted by Glimmer/Critica		
322	HPB8_1513	sodium/proline symporter	jhp0047	HP0055
323	HPB8_1524	hypothetical protein		
324	HPB8_1525	conserved hypothetical protein		HP1354
325	HPB8_1532	competence protein ComEC	jhp1279	HP1361
326	HPB8_1537	type III restriction-modification system: methylase		
327	HPB8_1543	UDP-N-acetylglucosamine acyltransferase	jhp1289	HP1375
328	HPB8_1550	hypothetical protein predicted by Glimmer/Critica		
329	HPB8_1553	conserved hypothetical protein	jhp1297	
330	HPB8_1554	hypothetical protein predicted by Glimmer/Critica		
331	HPB8_1557	hypothetical protein predicted by Glimmer/Critica		
332	HPB8_1558	hypothetical protein predicted by Glimmer/Critica		
333	HPB8_1559	hypothetical protein predicted by Glimmer/Critica		
334	HPB8_1560	hypothetical protein predicted by Glimmer/Critica		
335	HPB8_1565	hypothetical protein		
336	HPB8_1567	conserved hypothetical protein		HP0424
337	HPB8_1572	tRNA delta(2)-isopentenylpyrophosphate transferase	jhp1310	HP1415
338	HPB8_1574	adenine-specific methyltransferase EcoRI		
339	HPB8_1579	hypothetical protein predicted by Glimmer/Critica		
340	HPB8_1586	hypothetical protein predicted by Glimmer/Critica		
341	HPB8_1590	aspartate 1-decarboxylase	jhp0030	HP0034
342	HPB8_1591	ATP-dependent Clp protease ATP-binding subunit ClpA	jhp0029	HP0033
343	HPB8_1599	hypothetical protein predicted by Glimmer/Critica		
344	HPB8_1601	hypothetical protein predicted by Glimmer/Critica		
345	HPB8_1602	hypothetical protein predicted by Glimmer/Critica		
346	HPB8_1607	conserved hypothetical protein	jhp0016	HP0018
347	HPB8_1608	type IV secretion system protein VirB4	jhp0015	HP0017
348	HPB8_1612	tRNA(5-methylaminomethyl-2-thiouridylate)-methyl transferase	jhp0011	HP0013
349	HPB8_1617	hypothetical protein predicted by Glimmer/Critica		
350	HPB8_1620	orotidine-5'-phosphate decarboxylase	jhp0005	HP0005
351	HPB8_1625	conserved hypothetical protein		
352	HPB8_1628	UPF0174 protein jhp1494		
353	HPB8_1630	UPF0174 protein jhp1494		
354	HPB8_1632	hypothetical protein predicted by Glimmer/Critica		
355	HPB8_1636	hypothetical protein predicted by Glimmer/Critica		
356	HPB8_1637	hypothetical protein predicted by Glimmer/Critica		

Table S5 – continued from previous page

	coding sequence	product	J99-ortho	26695-ortho
357	HPB8_1638	undecaprenyl-phosphatealpha-N-acetylglucosaminyl transferase	jhp1488	HP1581
358	HPB8_1639	conserved hypothetical protein	jhp1486	HP1579
359	HPB8_1653	hypothetical protein predicted by Glimmer/Critica		
360	HPB8_1654	hypothetical protein predicted by Glimmer/Critica		
361	HPB8_1659	cell division protein FtsW	jhp1468	HP1560
362	HPB8_1660	hypothetical protein predicted by Glimmer/Critica		
363	HPB8_1664	conserved hypothetical protein	jhp1464	HP1556
364	HPB8_1665	conserved hypothetical protein		
365	HPB8_1672	conserved hypothetical protein	jhp1455	HP1544
366	HPB8_1675	leucyl-tRNA synthetase	jhp1452	HP1547
367	HPB8_1677	preprotein translocase SecF subunit	jhp1450	HP1549
368	HPB8_1681	putative ATP-dependent helicase	jhp1446	HP1553
369	HPB8_1690	conserved hypothetical protein		
370	HPB8_1695	hypothetical protein predicted by Glimmer/Critica		
371	HPB8_1704	type I restriction enzyme, R subunit	jhp1424	HP1402

Table S6: List of 54 genes of strain B8 with no 80/80 blastp hit in the proteome of strain B128 and without complete match with at most 2% differences on the DNA level. The genes in uncovered regions are shown in blue. The last two columns show the locus tags of all genes in strain J99 and strain 26695 which are ortholog to the given gene of strain B8.

	coding sequence	product	J99-ortho	26695-ortho
1	HPB8_81	hypothetical protein predicted by Glimmer/Critica		
2	HPB8_138	periplasmic protein TonB		
3	HPB8_193	hypothetical protein		
4	HPB8_237	blood group antigen-binding adhesin BabA fragment		
5	HPB8_238	hypothetical protein predicted by Glimmer/Critica		
6	HPB8_277	hypothetical protein predicted by Glimmer/Critica		
7	HPB8_386	hypothetical protein		
8	HPB8_389	conserved hypothetical protein		
9	HPB8_399	conserved hypothetical protein		HP1105
10	HPB8_427	Alpha-(1,3)-fucosyltransferase 11		
11	HPB8_428	fucosyltransferase		
12	HPB8_475	hypothetical protein		
13	HPB8_497	type IV secretion system protein VirB11		
14	HPB8_502	hypothetical protein predicted by Glimmer/Critica		
15	HPB8_519	conserved hypothetical protein, partial cds		
16	HPB8_529	conserved hypothetical protein		
17	HPB8_557	hypothetical protein predicted by Glimmer/Critica		
18	HPB8_582	Interferon-induced GTP-binding protein Mx2		HP0963
19	HPB8_583	hypothetical protein		
20	HPB8_626	conserved hypothetical protein		
21	HPB8_639	hypothetical protein predicted by Glimmer/Critica		
22	HPB8_655	Hydrogenase expression/formation protein hypD2		
23	HPB8_657	blood group antigen-binding adhesin BabA	jhp0833	HP1243
24	HPB8_658	hypothetical protein predicted by Glimmer/Critica		
25	HPB8_674	conserved hypothetical protein		
26	HPB8_692	Plasminogen-binding protein pgbA		
27	HPB8_716	cag pathogenicity island protein Y VirB10-like protein		
28	HPB8_733	GTP-binding protein Era	jhp0466	HP0517
29	HPB8_739	Regulator of nonsense transcripts 1		
30	HPB8_742	Regulator of nonsense transcripts 1 homolog		
31	HPB8_853	Chromosomal replication initiator protein dnaA		
32	HPB8_854	Alpha-(1,3)-fucosyltransferase 11		
33	HPB8_884	conserved hypothetical protein		
34	HPB8_888	ferrous iron transport protein B	jhp0627	HP0687
35	HPB8_922	conserved hypothetical protein	jhp0654	HP0716
36	HPB8_976	hypothetical protein predicted by Glimmer/Critica		

Table S6 – continued from previous page

	coding sequence	product	J99-ortho	26695-ortho
37	HPB8_998	type I restriction enzyme, S subunit		
38	HPB8_1057	type I restriction enzyme, S subunit		
39	HPB8_1072	plasminogen-binding protein pgbB	jhp0797	HP0863
40	HPB8_1092	conserved hypothetical protein		
41	HPB8_1093	hypothetical protein predicted by Glimmer/Critica		
42	HPB8_1104	conserved hypothetical protein	jhp0429	HP0477
43	HPB8_1447	conserved hypothetical protein		HP1187
44	HPB8_1467	methyl-accepting chemotaxis protein	jhp0091	HP0099
45	HPB8_1483	methyl-accepting chemotaxis protein	jhp0075	HP0082
46	HPB8_1521	adenine-specific DNA-methyltransferase	jhp0043	HP0050
47	HPB8_1522	putative agmatine deiminase		
48	HPB8_1561	hypothetical protein		
49	HPB8_1568	hypothetical protein		
50	HPB8_1576	agmatine deiminase	jhp0042	HP0049
51	HPB8_1618	hypothetical protein predicted by Glimmer/Critica		
52	HPB8_1680	Na+:H+ antiporter, NhaA family	jhp1447	HP1552
53	HPB8_1707	hypothetical protein predicted by Glimmer/Critica		
54	HPB8_1708	hypothetical protein predicted by Glimmer/Critica		

Table S7: List of 49 singletons of strain B128 when comparing it to strain B8. There is no functional annotation of the coding sequences available.

	coding sequence
1	HPB128_1g1
2	HPB128_2g10
3	HPB128_2g32
4	HPB128_3g47
5	HPB128_3g28
6	HPB128_6g1
7	HPB128_9g1
8	HPB128_11g29
9	HPB128_11g12
10	HPB128_16g66
11	HPB128_16g55
12	HPB128_19g22
13	HPB128_19g24
14	HPB128_19g25
15	HPB128_20g1
16	HPB128_21g168
17	HPB128_21g112
18	HPB128_21g120
19	HPB128_21g159
20	HPB128_21g242
21	HPB128_21g230
22	HPB128_22g1
23	HPB128_26g18
24	HPB128_65g4
25	HPB128_65g5
26	HPB128_129g1
27	HPB128_132g17
28	HPB128_142g38
29	HPB128_146g4
30	HPB128_146g1
31	HPB128_146g3
32	HPB128_146g2
33	HPB128_148g5
34	HPB128_148g8
35	HPB128_154g1
36	HPB128_155g47
37	HPB128_158g3
38	HPB128_161g1
39	HPB128_177g1
40	HPB128_179g2
41	HPB128_182g1
42	HPB128_182g4
43	HPB128_182g10
44	HPB128_184g1
45	HPB128_186g60
46	HPB128_187g16
47	HPB128_192g3
48	HPB128_199g87
49	HPB128_202g6

Table S8: List of 293 singletons of strain B8 when comparing it to strains J99, 26695, HPAG1, and P12.

	coding sequence	product
1	HPB8_60	hypothetical protein
2	HPB8_70	hypothetical protein predicted by Glimmer/Critica
3	HPB8_75	hypothetical protein predicted by Glimmer/Critica
4	HPB8_76	hypothetical protein predicted by Glimmer/Critica
5	HPB8_82	hypothetical protein predicted by Glimmer/Critica
6	HPB8_97	hypothetical protein predicted by Glimmer/Critica
7	HPB8_98	conserved hypothetical protein
8	HPB8_99	conserved hypothetical lipoprotein
9	HPB8_100	hypothetical protein predicted by Glimmer/Critica
10	HPB8_101	conserved hypothetical lipoprotein
11	HPB8_102	hypothetical protein
12	HPB8_103	hypothetical protein
13	HPB8_104	hypothetical protein predicted by Glimmer/Critica
14	HPB8_108	type I R-M system specificity subunit
15	HPB8_109	hypothetical protein predicted by Glimmer/Critica
16	HPB8_110	hypothetical protein predicted by Glimmer/Critica
17	HPB8_111	hypothetical protein predicted by Glimmer/Critica
18	HPB8_112	hypothetical protein predicted by Glimmer/Critica
19	HPB8_118	hypothetical protein
20	HPB8_119	conserved hypothetical protein
21	HPB8_127	Type II restriction enzyme RsrI
22	HPB8_128	hypothetical protein
23	HPB8_138	periplasmic protein TonB
24	HPB8_146	hypothetical protein predicted by Glimmer/Critica
25	HPB8_188	hypothetical protein predicted by Glimmer/Critica
26	HPB8_189	hypothetical protein predicted by Glimmer/Critica
27	HPB8_204	hypothetical protein predicted by Glimmer/Critica
28	HPB8_238	hypothetical protein predicted by Glimmer/Critica
29	HPB8_239	hypothetical protein predicted by Glimmer/Critica
30	HPB8_240	hypothetical protein predicted by Glimmer/Critica
31	HPB8_244	hypothetical protein predicted by Glimmer/Critica
32	HPB8_245	hypothetical protein predicted by Glimmer/Critica
33	HPB8_251	hypothetical protein predicted by Glimmer/Critica
34	HPB8_267	hypothetical protein predicted by Glimmer/Critica
35	HPB8_269	hypothetical protein predicted by Glimmer/Critica
36	HPB8_271	hypothetical protein predicted by Glimmer/Critica
37	HPB8_277	hypothetical protein predicted by Glimmer/Critica
38	HPB8_280	hypothetical protein

Table S8 – continued from previous page

	coding sequence	product
39	HPB8_282	hypothetical protein predicted by Glimmer/Critica
40	HPB8_293	hypothetical protein predicted by Glimmer/Critica
41	HPB8_298	hypothetical protein predicted by Glimmer/Critica
42	HPB8_305	hypothetical protein predicted by Glimmer/Critica
43	HPB8_307	hypothetical protein predicted by Glimmer/Critica
44	HPB8_315	conserved hypothetical protein
45	HPB8_316	conserved hypothetical protein
46	HPB8_317	hypothetical protein predicted by Glimmer/Critica
47	HPB8_318	hypothetical protein predicted by Glimmer/Critica
48	HPB8_323	hypothetical protein predicted by Glimmer/Critica
49	HPB8_335	hypothetical protein predicted by Glimmer/Critica
50	HPB8_339	Uncharacterized protein HI0977.
51	HPB8_340	Uncharacterized protein HI0977.
52	HPB8_344	hypothetical protein predicted by Glimmer/Critica
53	HPB8_347	hypothetical protein predicted by Glimmer/Critica
54	HPB8_355	hypothetical protein
55	HPB8_356	hypothetical protein
56	HPB8_357	hypothetical protein predicted by Glimmer/Critica
57	HPB8_383	conserved hypothetical protein
58	HPB8_386	hypothetical protein
59	HPB8_387	hypothetical protein
60	HPB8_389	conserved hypothetical protein
61	HPB8_428	fucosyltransferase
62	HPB8_434	hypothetical protein predicted by Glimmer/Critica
63	HPB8_443	putative chemotaxis protein
64	HPB8_444	chemotaxis protein
65	HPB8_455	hypothetical protein predicted by Glimmer/Critica
66	HPB8_462	hypothetical protein predicted by Glimmer/Critica
67	HPB8_475	hypothetical protein
68	HPB8_477	conserved hypothetical protein
69	HPB8_478	hypothetical protein predicted by Glimmer/Critica
70	HPB8_479	hypothetical protein predicted by Glimmer/Critica
71	HPB8_480	hypothetical protein predicted by Glimmer/Critica
72	HPB8_481	hypothetical protein predicted by Glimmer/Critica
73	HPB8_485	DNA transfer protein
74	HPB8_488	H. pylori predicted coding region HP0439
75	HPB8_489	Type IV secretion system protein virB8.
76	HPB8_490	component of conjugal plasmid transfer system
77	HPB8_491	hypothetical pseudogene
78	HPB8_492	DNA transformation compentancy
79	HPB8_493	DNA transformation compentancy

Table S8 – continued from previous page

	coding sequence	product
80	HPB8_500	hypothetical protein predicted by Glimmer/Critica
81	HPB8_502	hypothetical protein predicted by Glimmer/Critica
82	HPB8_506	conserved hypothetical protein
83	HPB8_508	hypothetical protein
84	HPB8_509	hypothetical protein
85	HPB8_510	conserved hypothetical protein
86	HPB8_514	hypothetical protein
87	HPB8_515	hypothetical protein
88	HPB8_516	hypothetical protein predicted by Glimmer/Critica
89	HPB8_517	conserved hypothetical protein, partial cds
90	HPB8_518	putative transposase
91	HPB8_519	conserved hypothetical protein, partial cds
92	HPB8_520	hypothetical protein predicted by Glimmer/Critica
93	HPB8_524	conserved hypothetical protein
94	HPB8_528	conserved hypothetical protein
95	HPB8_529	conserved hypothetical protein
96	HPB8_530	conserved hypothetical protein
97	HPB8_531	hypothetical protein predicted by Glimmer/Critica
98	HPB8_533	hypothetical protein
99	HPB8_543	conserved hypothetical protein
100	HPB8_547	hypothetical protein predicted by Glimmer/Critica
101	HPB8_552	conserved hypothetical protein
102	HPB8_557	hypothetical protein predicted by Glimmer/Critica
103	HPB8_559	hypothetical protein predicted by Glimmer/Critica
104	HPB8_560	hypothetical protein predicted by Glimmer/Critica
105	HPB8_562	hypothetical protein predicted by Glimmer/Critica
106	HPB8_563	hypothetical protein predicted by Glimmer/Critica
107	HPB8_564	hypothetical protein predicted by Glimmer/Critica
108	HPB8_565	hypothetical protein predicted by Glimmer/Critica
109	HPB8_570	hypothetical protein predicted by Glimmer/Critica
110	HPB8_581	Probable tRNA modification GTPase trmE.
111	HPB8_583	hypothetical protein
112	HPB8_589	hypothetical protein predicted by Glimmer/Critica
113	HPB8_599	hypothetical protein predicted by Glimmer/Critica
114	HPB8_600	hypothetical protein predicted by Glimmer/Critica
115	HPB8_601	hypothetical protein predicted by Glimmer/Critica
116	HPB8_611	conserved hypothetical protein
117	HPB8_612	hypothetical protein predicted by Glimmer/Critica
118	HPB8_626	conserved hypothetical protein
119	HPB8_633	hypothetical protein predicted by Glimmer/Critica
120	HPB8_634	Ferric enterobactin receptor precursor.

Table S8 – continued from previous page

	coding sequence	product
121	HPB8_635	hypothetical protein predicted by Glimmer/Critica
122	HPB8_639	hypothetical protein predicted by Glimmer/Critica
123	HPB8_640	hypothetical protein predicted by Glimmer/Critica
124	HPB8_655	Hydrogenase expression/formation protein hypD2.
125	HPB8_658	hypothetical protein predicted by Glimmer/Critica
126	HPB8_660	Uncharacterized protein HI0711.
127	HPB8_665	Vacuolating cytotoxin precursor.
128	HPB8_666	Vacuolating cytotoxin precursor.
129	HPB8_671	conserved hypothetical protein
130	HPB8_688	hypothetical protein
131	HPB8_689	hypothetical protein
132	HPB8_692	Plasminogen-binding protein pgbA.
133	HPB8_695	hypothetical protein predicted by Glimmer/Critica
134	HPB8_707	hypothetical protein predicted by Glimmer/Critica
135	HPB8_716	cag pathogenicity island protein Y VirB10-like protein
136	HPB8_720	hypothetical protein predicted by Glimmer/Critica
137	HPB8_721	hypothetical protein predicted by Glimmer/Critica
138	HPB8_727	hypothetical protein predicted by Glimmer/Critica
139	HPB8_735	conserved hypothetical protein
140	HPB8_736	hypothetical protein predicted by Glimmer/Critica
141	HPB8_737	hypothetical protein predicted by Glimmer/Critica
142	HPB8_738	hypothetical protein predicted by Glimmer/Critica
143	HPB8_739	Regulator of nonsense transcripts 1
144	HPB8_740	hypothetical protein predicted by Glimmer/Critica
145	HPB8_751	hypothetical protein predicted by Glimmer/Critica
146	HPB8_752	hypothetical protein predicted by Glimmer/Critica
147	HPB8_759	hypothetical protein predicted by Glimmer/Critica
148	HPB8_777	hypothetical protein
149	HPB8_798	Phosphate import ATP-binding protein pstB
150	HPB8_827	hypothetical protein predicted by Glimmer/Critica
151	HPB8_830	hypothetical protein predicted by Glimmer/Critica
152	HPB8_840	hypothetical protein predicted by Glimmer/Critica
153	HPB8_853	Chromosomal replication initiator protein dnaA.
154	HPB8_854	Alpha-(1,3)-fucosyltransferase 11
155	HPB8_872	NAD-dependent deacetylase
156	HPB8_873	hypothetical protein predicted by Glimmer/Critica
157	HPB8_875	hypothetical protein predicted by Glimmer/Critica
158	HPB8_877	conserved hypothetical protein
159	HPB8_895	5-oxoprolinase
160	HPB8_899	hypothetical protein predicted by Glimmer/Critica
161	HPB8_905	hypothetical protein predicted by Glimmer/Critica

Table S8 – continued from previous page

	coding sequence	product
162	HPB8_906	hypothetical protein predicted by Glimmer/Critica
163	HPB8_907	conserved hypothetical protein
164	HPB8_908	conserved hypothetical protein
165	HPB8_911	hypothetical protein predicted by Glimmer/Critica
166	HPB8_917	hypothetical protein
167	HPB8_918	hypothetical protein
168	HPB8_919	hypothetical protein
169	HPB8_936	hypothetical protein
170	HPB8_937	Dynactin subunit 1
171	HPB8_938	hypothetical protein
172	HPB8_939	conserved hypothetical protein
173	HPB8_951	conserved hypothetical protein
174	HPB8_708	cag pathogenicity island protein R
175	HPB8_7	site-specific DNA-methyltransferase (adenine-specific)
176	HPB8_8	adenine specific DNA methyltransferase
177	HPB8_11	hypothetical protein
178	HPB8_12	conserved hypothetical protein
179	HPB8_13	conserved hypothetical protein
180	HPB8_16	hypothetical protein predicted by Glimmer/Critica
181	HPB8_58	conserved hypothetical protein
182	HPB8_59	conserved hypothetical protein
183	HPB8_973	conserved hypothetical protein
184	HPB8_975	hypothetical protein predicted by Glimmer/Critica
185	HPB8_976	hypothetical protein predicted by Glimmer/Critica
186	HPB8_997	hypothetical protein predicted by Glimmer/Critica
187	HPB8_998	type I restriction enzyme, S subunit
188	HPB8_1028	hypothetical protein predicted by Glimmer/Critica
189	HPB8_1042	hypothetical protein predicted by Glimmer/Critica
190	HPB8_1046	hypothetical protein predicted by Glimmer/Critica
191	HPB8_1057	type I restriction enzyme, S subunit
192	HPB8_1059	DNA (cytosine-5-)-methyltransferase
193	HPB8_1060	type II restriction endonuclease
194	HPB8_1065	hypothetical protein predicted by Glimmer/Critica
195	HPB8_1080	hypothetical protein predicted by Glimmer/Critica
196	HPB8_1081	CDP-diacylglycerol phosphotidylhydrolase
197	HPB8_1082	CDP-diacylglycerol phosphotidylhydrolase
198	HPB8_1083	hypothetical protein predicted by Glimmer/Critica
199	HPB8_1091	hypothetical protein predicted by Glimmer/Critica
200	HPB8_1092	conserved hypothetical protein
201	HPB8_1093	hypothetical protein predicted by Glimmer/Critica
202	HPB8_1100	DNA adenine methylase

Table S8 – continued from previous page

	coding sequence	product
203	HPB8_1101	adenine specific DNA methyltransferase
204	HPB8_1103	site-specific DNA-methyltransferase (adenine-specific)
205	HPB8_1105	hypothetical protein predicted by Glimmer/Critica
206	HPB8_1119	type I restriction enzyme R protein
207	HPB8_1120	type I restriction enzyme R protein
208	HPB8_1123	type I restriction enzyme S protein
209	HPB8_1124	hypothetical protein predicted by Glimmer/Critica
210	HPB8_1125	hypothetical protein predicted by Glimmer/Critica
211	HPB8_1137	hypothetical protein predicted by Glimmer/Critica
212	HPB8_1138	hypothetical protein predicted by Glimmer/Critica
213	HPB8_1139	hypothetical protein
214	HPB8_1140	hypothetical protein
215	HPB8_1168	hypothetical protein predicted by Glimmer/Critica
216	HPB8_1190	hypothetical protein predicted by Glimmer/Critica
217	HPB8_1222	hypothetical protein predicted by Glimmer/Critica
218	HPB8_1226	conserved hypothetical protein
219	HPB8_1228	hypothetical protein predicted by Glimmer/Critica
220	HPB8_1229	Hcp beta-lactamase-like protein C1orf163.
221	HPB8_1242	hypothetical protein predicted by Glimmer/Critica
222	HPB8_1243	conserved hypothetical protein
223	HPB8_1248	hypothetical protein predicted by Glimmer/Critica
224	HPB8_1300	Uncharacterized protein HP0262.
225	HPB8_1301	Uncharacterized protein HP0262.
226	HPB8_1302	conserved hypothetical protein
227	HPB8_1311	hypothetical protein predicted by Glimmer/Critica
228	HPB8_1329	hypothetical protein predicted by Glimmer/Critica
229	HPB8_1330	hypothetical protein predicted by Glimmer/Critica
230	HPB8_1339	hypothetical protein predicted by Glimmer/Critica
231	HPB8_1340	hypothetical protein predicted by Glimmer/Critica
232	HPB8_1355	hypothetical protein predicted by Glimmer/Critica
233	HPB8_1363	hypothetical protein predicted by Glimmer/Critica
234	HPB8_1380	hypothetical protein predicted by Glimmer/Critica
235	HPB8_1404	hypothetical protein predicted by Glimmer/Critica
236	HPB8_1432	hypothetical protein predicted by Glimmer/Critica
237	HPB8_1435	hypothetical protein predicted by Glimmer/Critica
238	HPB8_1437	hypothetical protein predicted by Glimmer/Critica
239	HPB8_1445	Uncharacterized protein jhp0112; Flags: Precursor;
240	HPB8_1465	hypothetical protein predicted by Glimmer/Critica
241	HPB8_1486	hypothetical protein predicted by Glimmer/Critica
242	HPB8_1491	hypothetical protein predicted by Glimmer/Critica
243	HPB8_1500	hypothetical protein

Table S8 – continued from previous page

	coding sequence	product
244	HPB8_1504	conserved hypothetical protein
245	HPB8_1508	Chaperone protein dnaK
246	HPB8_1509	hypothetical protein predicted by Glimmer/Critica
247	HPB8_1510	hypothetical protein
248	HPB8_1515	hypothetical protein predicted by Glimmer/Critica
249	HPB8_1517	adenine specific DNA methylase (Mod-related)
250	HPB8_1518	DNA methyltransferase
251	HPB8_1522	Putative agmatine deiminase
252	HPB8_1524	hypothetical protein
253	HPB8_1538	Modification methylase KpnI
254	HPB8_1546	hypothetical protein predicted by Glimmer/Critica
255	HPB8_1550	hypothetical protein predicted by Glimmer/Critica
256	HPB8_1552	adenine-specific DNA-methyltransferase
257	HPB8_1554	hypothetical protein predicted by Glimmer/Critica
258	HPB8_1559	hypothetical protein predicted by Glimmer/Critica
259	HPB8_1560	hypothetical protein predicted by Glimmer/Critica
260	HPB8_1561	hypothetical protein
261	HPB8_1562	hypothetical protein
262	HPB8_1563	hypothetical protein
263	HPB8_1564	hypothetical protein
264	HPB8_1565	hypothetical protein
265	HPB8_1566	hypothetical protein
266	HPB8_1568	hypothetical protein
267	HPB8_1569	hypothetical protein
268	HPB8_1575	adenine DNA methyltransferase protein
269	HPB8_1579	hypothetical protein predicted by Glimmer/Critica
270	HPB8_1594	conserved hypothetical protein
271	HPB8_1599	hypothetical protein predicted by Glimmer/Critica
272	HPB8_1601	hypothetical protein predicted by Glimmer/Critica
273	HPB8_1602	hypothetical protein predicted by Glimmer/Critica
274	HPB8_1617	hypothetical protein predicted by Glimmer/Critica
275	HPB8_1618	hypothetical protein predicted by Glimmer/Critica
276	HPB8_1625	conserved hypothetical protein
277	HPB8_1628	UPF0174 protein jhp1494;
278	HPB8_1630	UPF0174 protein jhp1494.
279	HPB8_1632	hypothetical protein predicted by Glimmer/Critica
280	HPB8_1636	hypothetical protein predicted by Glimmer/Critica
281	HPB8_1637	hypothetical protein predicted by Glimmer/Critica
282	HPB8_1653	hypothetical protein predicted by Glimmer/Critica
283	HPB8_1654	hypothetical protein predicted by Glimmer/Critica
284	HPB8_1660	hypothetical protein predicted by Glimmer/Critica

Table S8 – continued from previous page

	coding sequence	product
285	HPB8_1665	conserved hypothetical protein
286	HPB8_1684	hypothetical protein
287	HPB8_1685	hypothetical protein
288	HPB8_1690	conserved hypothetical protein
289	HPB8_1695	hypothetical protein predicted by Glimmer/Critica
290	HPB8_1697	hypothetical protein
291	HPB8_1699	hypothetical protein
292	HPB8_1706	putative type I restriction enzyme (specificity subunit)
293	HPB8_1708	hypothetical protein predicted by Glimmer/Critica

Table S9: List of 35 singletons of strain B8 versus J99 and P12, i.e. the list of genes in strain B8 that have neither an ortholog in strain J99 nor in P12 and which do not appear in Table S8. The last column shows all genes in strain 26695 which are ortholog to the given B8-gene.

	coding sequence	product	26695-ortho
1	HPB8_30	DNA adenine methylase	
2	HPB8_31	putative type II methylase	
3	HPB8_155	conserved hypothetical protein	HP1324
4	HPB8_193	hypothetical protein	
5	HPB8_279	ulcer-associated gene restriction endonuclease	
6	HPB8_399	conserved hypothetical protein	HP1105
7	HPB8_484	conserved hypothetical protein	HP0444
8	HPB8_486	Protein virB4 precursor	HP0441
9	HPB8_487	DNA topoisomerase I	HP0440
10	HPB8_521	conserved hypothetical protein	HP0995
11	HPB8_558	conserved hypothetical protein	HP0982
12	HPB8_580	conserved hypothetical protein	HP0966
13	HPB8_582	Interferon-induced GTP-binding protein Mx2	HP0963
14	HPB8_610	conserved hypothetical protein	HP0938
15	HPB8_632	probable hemoglobin and hemoglobin-haptoglobin-binding protein 4 precursor	HP0916
16	HPB8_656	conserved hypothetical protein	HP0897
17	HPB8_674	conserved hypothetical protein	
18	HPB8_724	cag pathogenicity island protein Epsilon	
19	HPB8_790	putative glycosyl transferase	
20	HPB8_791	hypothetical protein	
21	HPB8_930	sialic acid-binding adhesin SabA	HP0725
22	HPB8_963	hypothetical protein	HP0756
23	HPB8_972	hypothetical protein	
24	HPB8_974	conserved hypothetical protein	
25	HPB8_1097	conserved hypothetical protein	HP0484
26	HPB8_1121	type I restriction enzyme, R subunit	
27	HPB8_1213	conserved hypothetical protein	
28	HPB8_1338	conserved hypothetical protein	HP0227
29	HPB8_1439	hypothetical protein	HP0128
30	HPB8_1447	conserved hypothetical protein	HP1187
31	HPB8_1474	HrgA protein	
32	HPB8_1507	conserved hypothetical protein	HP0060
33	HPB8_1514	adenine/cytosine DNA methyltransferase	
34	HPB8_1525	conserved hypothetical protein	HP1354
35	HPB8_1537	type III restriction-modification system: methylase	

Table S10: List of 74 singletons of strain B8 versus 26695 and HPAG1, i.e. the list of genes in strain B8 that have neither an ortholog in strain 26695 nor in HPAG1 and which do not appear in Table S8. The last column shows all genes in strain J99 which are ortholog to the given B8-gene.

	coding sequence	product	J99-ortho
1	HPB8_10	putative type IIS restriction/modification enzyme	
2	HPB8_81	hypothetical protein predicted by Glimmer/Critica	
3	HPB8_117	hypothetical protein predicted by Glimmer/Critica	
4	HPB8_266	hypothetical protein predicted by Glimmer/Critica	
5	HPB8_299	secreted protein involved in flagellar motility	jhp1117
6	HPB8_320	hypothetical protein predicted by Glimmer/Critica	
7	HPB8_358	conserved hypothetical protein	
8	HPB8_360	conserved hypothetical protein	
9	HPB8_388	hypothetical protein	jhp1043
10	HPB8_416	conserved hypothetical protein	jhp1013
11	HPB8_483	conserved hypothetical protein	
12	HPB8_494	conserved hypothetical protein	
13	HPB8_495	conserved hypothetical protein	
14	HPB8_496	conserved hypothetical protein	
15	HPB8_497	type IV secretion system protein VirB11	
16	HPB8_498	conserved hypothetical protein	
17	HPB8_499	conserved hypothetical protein	
18	HPB8_501	conserved hypothetical protein	
19	HPB8_507	hypothetical protein predicted by Glimmer/Critica	
20	HPB8_511	conserved hypothetical protein	jhp0945
21	HPB8_512	conserved hypothetical protein	jhp0947
22	HPB8_513	conserved hypothetical protein	jhp0948
23	HPB8_525	conserved hypothetical protein	jhp0939
24	HPB8_526	conserved hypothetical protein	jhp0938
25	HPB8_527	type IV secretion system protein TrbL	jhp0937
26	HPB8_532	conserved hypothetical protein	jhp0935
27	HPB8_534	hypothetical protein	
28	HPB8_535	conserved hypothetical protein	jhp0933
29	HPB8_536	conserved hypothetical protein	jhp0932
30	HPB8_537	DNA topoisomerase I	jhp0931
31	HPB8_538	type IV secretion system protein VirD4	
32	HPB8_539	conserved hypothetical protein	
33	HPB8_540	type IV secretion system protein VirB11	
34	HPB8_541	conserved hypothetical protein	
35	HPB8_542	conserved hypothetical protein	
36	HPB8_544	type IV secretion system protein VirB10	
37	HPB8_545	type IV secretion system protein VirB9	

Table S10 – continued from previous page

	coding sequence	product	J99-ortho
38	HPB8_546	putative VirB8 protein	
39	HPB8_551	conserved hypothetical protein	
40	HPB8_553	conserved hypothetical protein	
41	HPB8_554	conserved hypothetical protein	
42	HPB8_556	integrase/recombinase (XerCD family)	jhp0951
43	HPB8_647	phosphate acetyltransferase	jhp0841
44	HPB8_648	Acetate kinase	
45	HPB8_649	Acetate kinase	
46	HPB8_650	Acetate kinase	
47	HPB8_729	conserved hypothetical protein	
48	HPB8_809	putative ABC-2 type transport system permease protein	jhp0299
49	HPB8_811	hypothetical protein predicted by Glimmer/Critica	
50	HPB8_818	jhp0563-like glycosyltransferase, family 25	
51	HPB8_842	conserved hypothetical protein	jhp0584
52	HPB8_884	conserved hypothetical protein	
53	HPB8_896	RBL01243	
54	HPB8_927	Mesentericin Y105 secretion protein mesE	jhp1103
55	HPB8_940	hypothetical protein	
56	HPB8_1016	holo-[acyl-carrier protein] synthase	
57	HPB8_1098	DNA (cytosine-5-)-methyltransferase	jhp0435
58	HPB8_1110	hypothetical protein predicted by Glimmer/Critica	
59	HPB8_1151	conserved hypothetical protein	
60	HPB8_1197	conserved hypothetical protein	
61	HPB8_1198	conserved hypothetical protein	jhp0346
62	HPB8_1212	hypothetical protein predicted by Glimmer/Critica	
63	HPB8_1361	conserved hypothetical protein	
64	HPB8_1379	conserved hypothetical protein	jhp0174
65	HPB8_1485	conserved hypothetical protein	jhp0073
66	HPB8_1494	hypothetical protein predicted by Glimmer/Critica	
67	HPB8_1516	conserved hypothetical protein	jhp0046
68	HPB8_1519	conserved hypothetical protein	
69	HPB8_1523	hypothetical protein	
70	HPB8_1574	adenine-specific methyltransferase EcoRI	
71	HPB8_1627	hypothetical protein predicted by Glimmer/Critica	
72	HPB8_1629	UPF0174 protein HP1587	
73	HPB8_1698	hypothetical protein	
74	HPB8_1707	hypothetical protein predicted by Glimmer/Critica	

Table S11: List of 84 coding sequences in the plasticity zone of strain B8. The last two columns show the locus tags of all genes in strain J99 and strain 26695 which are ortholog to the given gene of strain B8.

	coding sequence	product	J99-ortho	26695-ortho
1	HPB8_481	hypothetical protein predicted by Glimmer/Critica		
2	HPB8_482	conserved hypothetical protein		HP0446
3	HPB8_483	conserved hypothetical protein		
4	HPB8_484	conserved hypothetical protein		HP0444
5	HPB8_485	DNA transfer protein		
6	HPB8_486	Protein virB4 precursor.		HP0441
7	HPB8_487	DNA topoisomerase I		HP0440
8	HPB8_488	H. pylori predicted coding region HP0439		
9	HPB8_489	Type IV secretion system protein virB8.		
10	HPB8_490	component of conjugal plasmid transfer system		
11	HPB8_491	hypothetical pseudogene		
12	HPB8_492	DNA transformation compentancy		
13	HPB8_493	DNA transformation compentancy		
14	HPB8_494	conserved hypothetical protein		
15	HPB8_495	conserved hypothetical protein		
16	HPB8_496	conserved hypothetical protein		
17	HPB8_497	type IV secretion system protein VirB11		
18	HPB8_498	conserved hypothetical protein		
19	HPB8_499	hypothetical protein		
20	HPB8_500	hypothetical protein predicted by Glimmer/Critica		
21	HPB8_501	conserved hypothetical protein		
22	HPB8_502	hypothetical protein predicted by Glimmer/Critica		
23	HPB8_503	conserved hypothetical protein		HP1002
24	HPB8_504	conserved hypothetical protein		HP1001
25	HPB8_505	putative chromosome partitioning protein		HP1000
26	HPB8_506	conserved hypothetical protein		
27	HPB8_507	hypothetical protein predicted by Glimmer/Critica		
28	HPB8_508	hypothetical protein		
29	HPB8_509	hypothetical protein		
30	HPB8_510	conserved hypothetical protein		
31	HPB8_511	conserved hypothetical protein	jhp0945	
32	HPB8_512	conserved hypothetical protein	jhp0947	
33	HPB8_513	conserved hypothetical protein	jhp0948	
34	HPB8_514	hypothetical protein		
35	HPB8_515	hypothetical protein		
36	HPB8_516	hypothetical protein predicted by Glimmer/Critica		
37	HPB8_517	conserved hypothetical protein, partial cds		

	coding sequence	product	J99-ortho	26695-ortho
38	HPB8_518	putative transposase		
39	HPB8_519	conserved hypothetical protein, partial cds		
40	HPB8_520	hypothetical protein predicted by Glimmer/Critica		
41	HPB8_521	conserved hypothetical protein		HP0995
42	HPB8_522	conserved hypothetical protein		HP0994
43	HPB8_523	conserved hypothetical protein		HP0993
44	HPB8_524	conserved hypothetical protein		
45	HPB8_525	conserved hypothetical protein	jhp0939	
46	HPB8_526	conserved hypothetical protein	jhp0938	
47	HPB8_527	type IV secretion system protein TrbL	jhp0937	
48	HPB8_528	conserved hypothetical protein		
49	HPB8_529	conserved hypothetical protein		
50	HPB8_530	conserved hypothetical protein		
51	HPB8_531	hypothetical protein predicted by Glimmer/Critica		
52	HPB8_532	conserved hypothetical protein	jhp0935	
53	HPB8_533	hypothetical protein		
54	HPB8_534	hypothetical protein		
55	HPB8_535	conserved hypothetical protein	jhp0933	
56	HPB8_536	conserved hypothetical protein	jhp0932	
57	HPB8_537	DNA topoisomerase I	jhp0931	
58	HPB8_538	type IV secretion system protein VirD4		
59	HPB8_539	conserved hypothetical protein		
60	HPB8_540	type IV secretion system protein VirB11		
61	HPB8_541	conserved hypothetical protein		
62	HPB8_542	conserved hypothetical protein		
63	HPB8_543	conserved hypothetical protein		
64	HPB8_544	type IV secretion system protein VirB10		
65	HPB8_545	type IV secretion system protein VirB9		
66	HPB8_546	putative VirB8 protein		
67	HPB8_547	hypothetical protein predicted by Glimmer/Critica		
68	HPB8_548	type IV secretion system protein VirB4		HP0459
69	HPB8_549	conserved hypothetical protein		HP0458
70	HPB8_550	conserved hypothetical protein		HP0457
71	HPB8_551	conserved hypothetical protein		
72	HPB8_552	conserved hypothetical protein		
73	HPB8_553	conserved hypothetical protein		
74	HPB8_554	conserved hypothetical protein		
75	HPB8_555	conserved hypothetical protein	jhp0950	
76	HPB8_556	integrase/recombinase (XerCD family)	jhp0951	
77	HPB8_557	hypothetical protein predicted by Glimmer/Critica		
78	HPB8_558	conserved hypothetical protein		HP0982

Table S11 – continued from previous page

	coding sequence	product	J99-ortho	26695-ortho
79	HPB8_559	hypothetical protein predicted by Glimmer/Critica		
80	HPB8_560	hypothetical protein predicted by Glimmer/Critica		
81	HPB8_561	small conductance mechanosensitive ion channel, MscS family	jhp0915	HP0983
82	HPB8_562	hypothetical protein predicted by Glimmer/Critica		
83	HPB8_563	hypothetical protein predicted by Glimmer/Critica		
84	HPB8_564	hypothetical protein predicted by Glimmer/Critica		

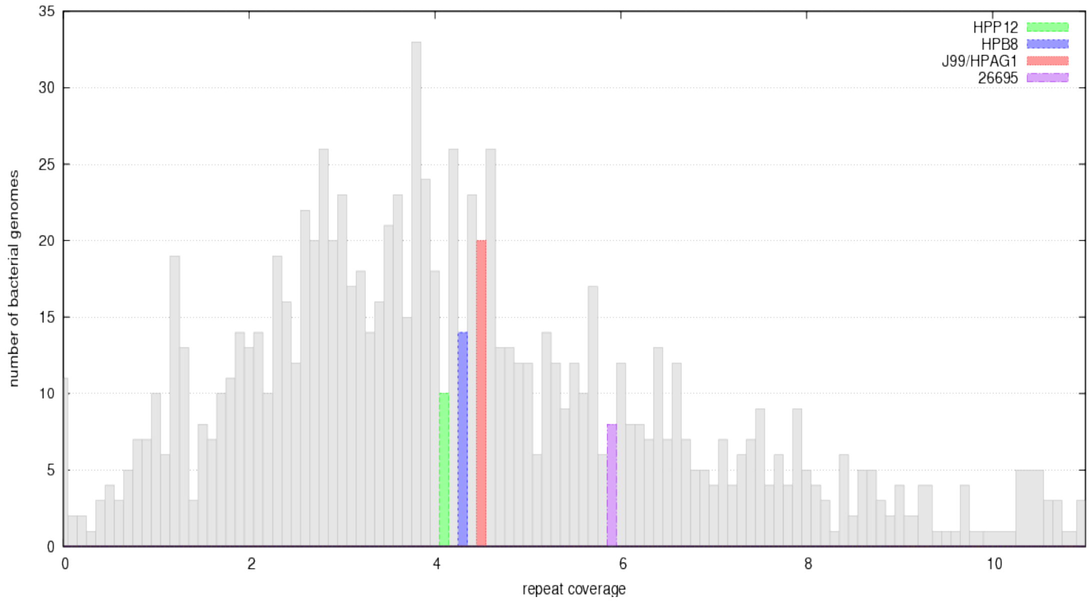

Figure S1: Distribution of the repeat density for 1053 bacterial genomes. These were downloaded from `http://www.ebi.ac.uk/genomes/bacteria.html`. All genome sequences of less than 100,000 bp where eliminated from this comparison. For each genome we computed the maximal repeats of length at least 100 bp, with at least 80% sequence identity. The repeat density is the ratio of the number of bp covered by these repeats and the genome length. The x-axes shows the repeat densities and the y-axes shows how many bacterial genomes have the given repeat density. For example, there are 33 baterial genomes with a repeat density of 3.8%. The complete distribution ranges from 0% to 35.9%, but only few genomes have a repeat density larger than 11%. Therefore, the distribution is only shown for the range from 0% to 11%. To show where the repeat densities of the different *H. pylori* genomes are, we use different colors for the corresponding density columns. For example, the genome of strain B8 and 13 other genomes have a repeat density of 4.3%, which is depicted in blue.

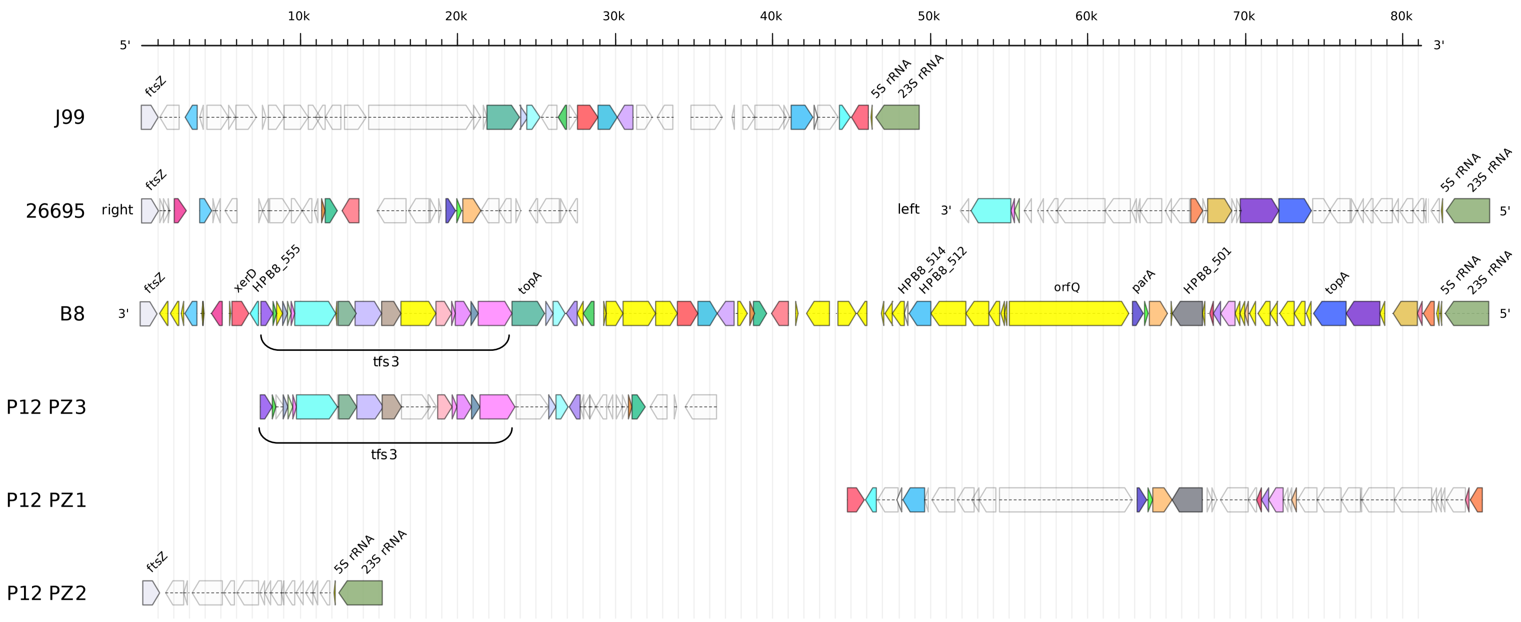

Figure S2: Comparison of genes in the plasticity zones of four *Helicobacter pylori* strains (J99, 26695, B8 and P12). Depicted gene regions are chosen according to Table 1,except for the flanking *ftsZ* and rRNA genes which are additionally displayed. Genes with significant similarity are drawn in the same color. Genes in the PZ of strain B8 with no significant similarity to genes in the plasticity zones of the other strains are highlighted in yellow. Those singletons in the other three strains are drawn in faded grey. To improve clearness, the B8 plasticity zone and the 26695 left PZ is drawn in inverted direction (from 3’ to 5’). All genes labeled on the B8 plasticity zone refer to features mentioned in the text.

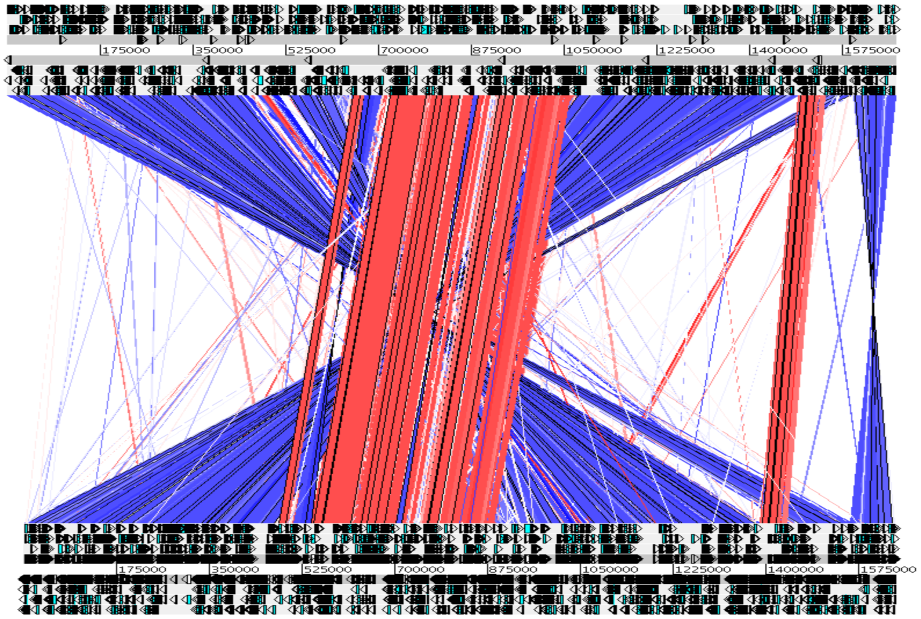

Figure S3: Synteny plot of strain B8 (top) versus strain J99 (bottom).

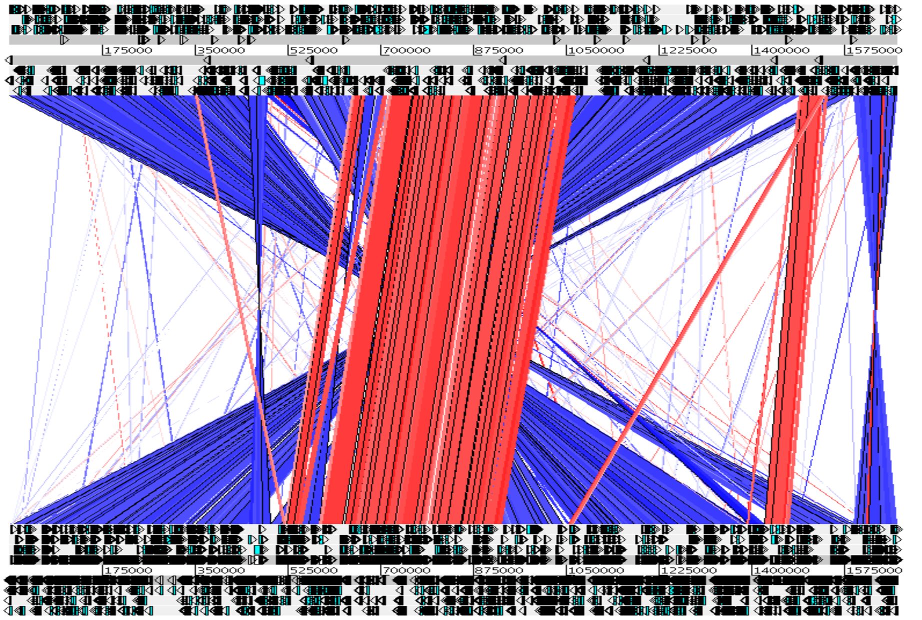

Figure S4: Synteny plot of strain B8 (top) versus strain P12 (bottom).

A

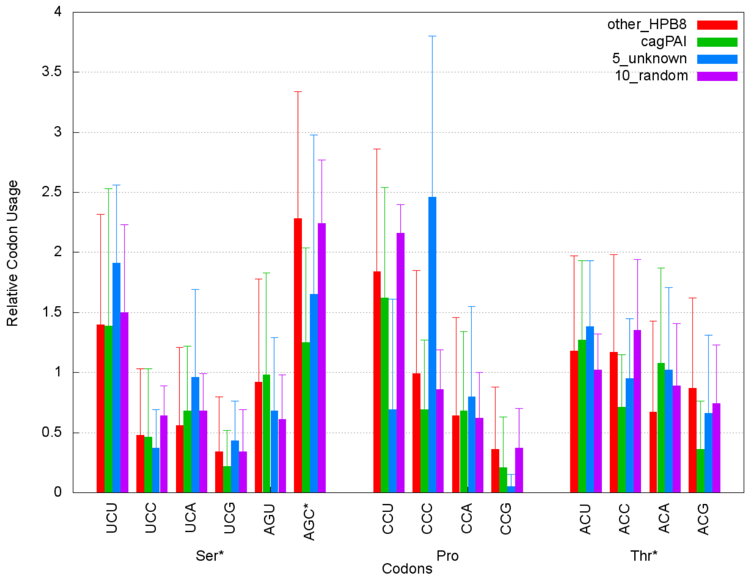

B

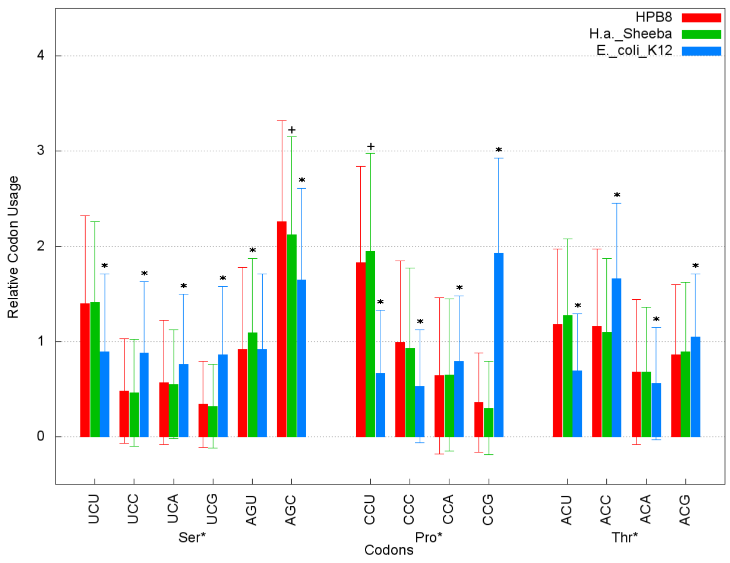

Figure S5: The relative codon usage was calculated per gene and then averaged per group. Error bars indicate the standard deviation. All stared amino acids have differences with $p < 0.01$ (99% confidence) according to an ANOVA. The figures show the results for the codons translating to Serine, Proline, and Threonine. The results for the remaining codons are shown in Figure S6 and Figure S7. **(A)**: Relative codon usage comparison of (a) the coding sequences from HPB8_735 to HPB8_739, (b) the *cag*-PAI genes, (c) all other genes of strain B8, and (d) ten coding sequence randomly selected from strain B8. Codons in which there is a difference between *cag*PAI and the rest of the genome are marked with a star. Due to the small sample size of the randomly chosen genes and the 5 unknown genes, there were no significant differences detectable between these and other groups. **(B)**: Relative codon usage comparison of strain B8, *H. acinonychis* Sheeba and *E. coli* K12. Symbols above the error bars indicate statistically identical groups within one codon. Codons with a star only above one of the bars have a mean that is significantly different from the two codons without a star (two groups). For codons with two symbols, all means are statistically different to each other (3 groups). Groups in **(A)** and **(B)** were found using the TukeyHSD post-hoc test.

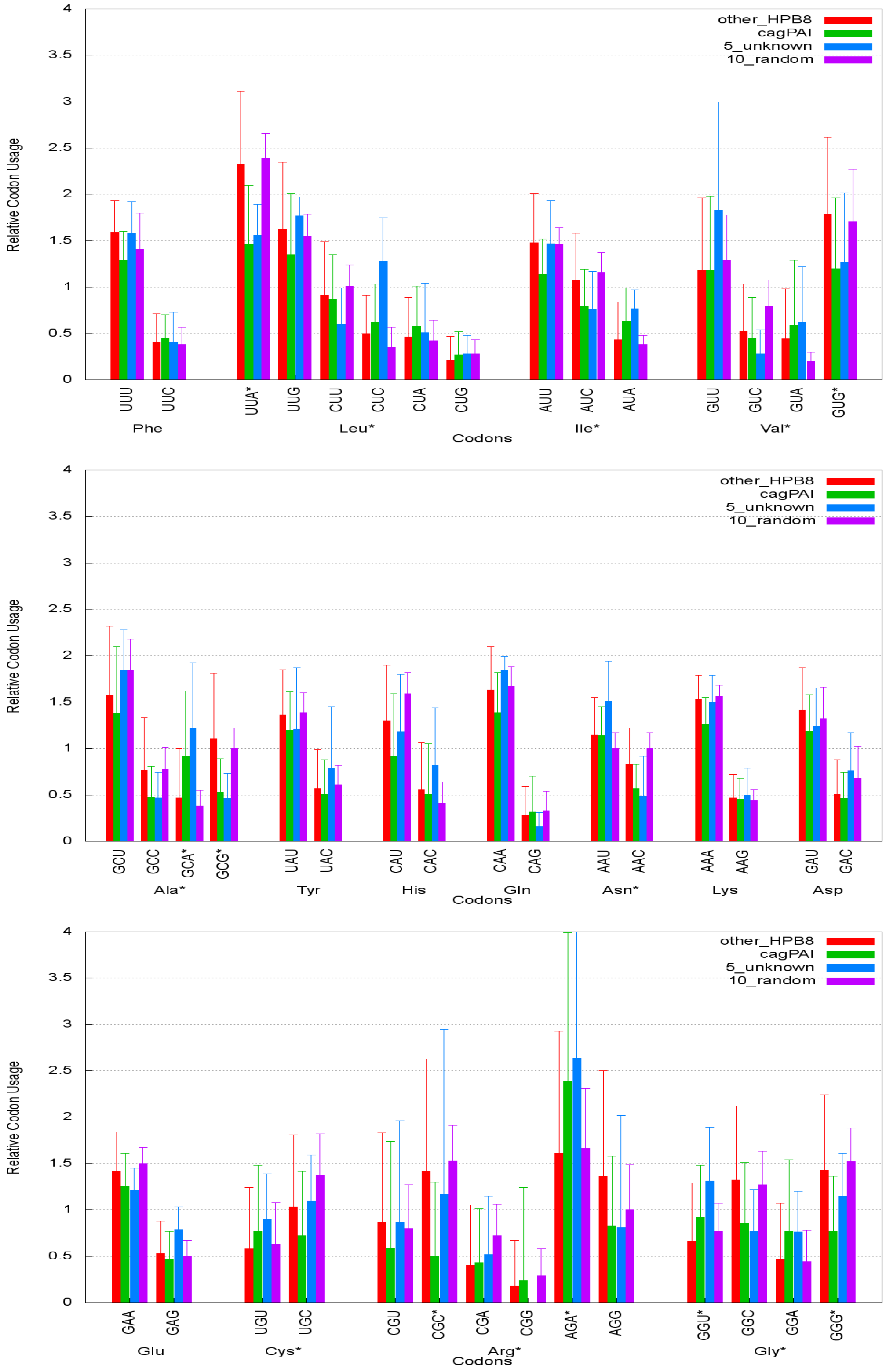

Figure S6: Codon usage of four groups of B8 genes for the codons translating to amino acids different from Serine, Proline, and Threonine.

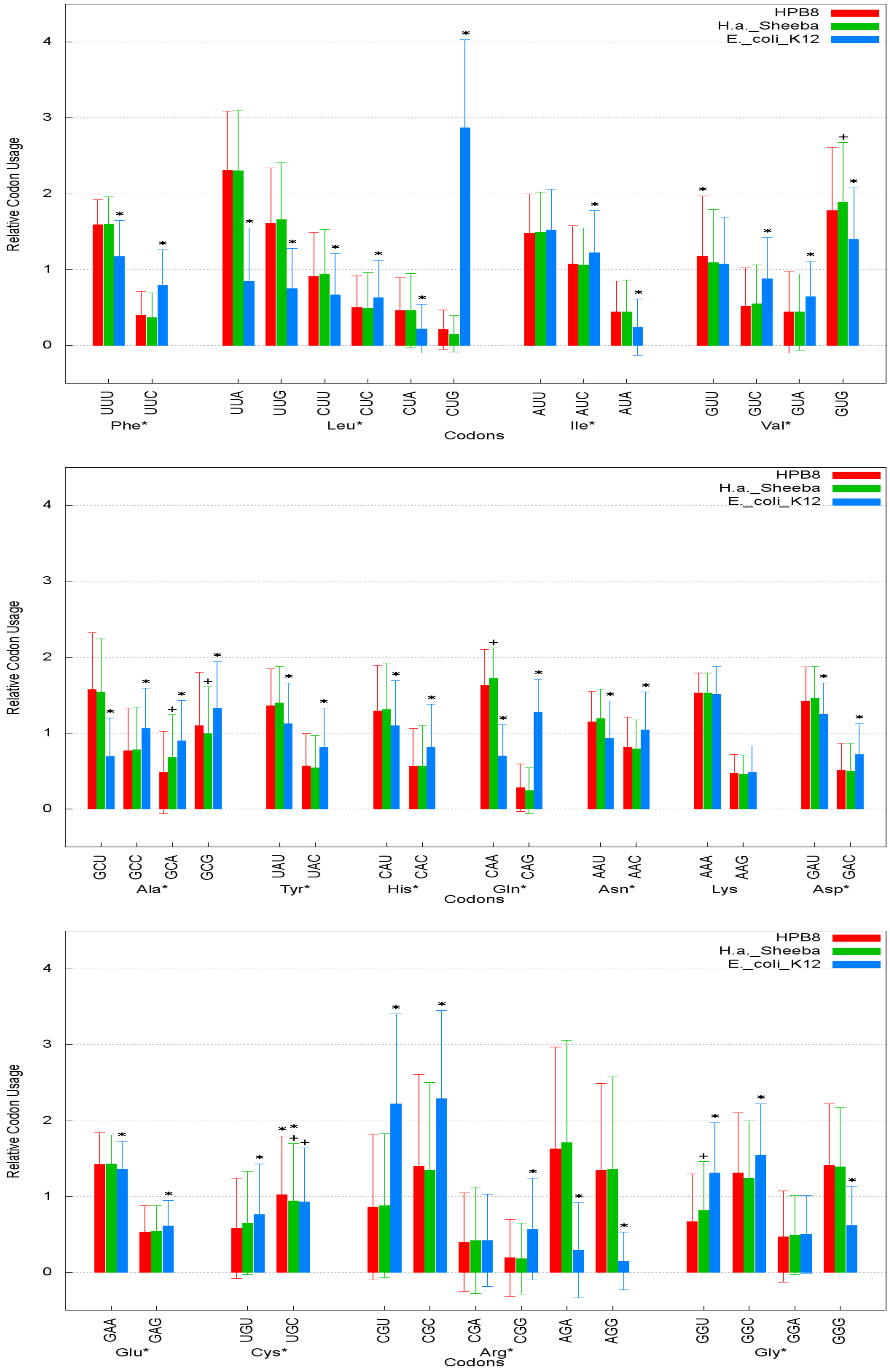

Figure S7: Codon usage of strain B8, *H. acinonychis* Sheeba, and *E. coli* K12 for the codons translating to amino acids different from Serine, Proline, and Threonine.
